# Preventive Versus Curative Application of Endophytic *Trichoderma harzianum* and *Bacillus subtilis* for Biological Control of *Meloidogyne javanica* and *M. incognita* in Olive Under Greenhouse Conditions

**DOI:** 10.3390/pathogens15060624

**Published:** 2026-06-11

**Authors:** Salah-Eddine Laasli, Abdelfattah A. Dababat, Fouad Mokrini, Elhassan Mayad, Amal Hari, Honglian Li, Hajar El Hamss, Mustafa İmren, Muhammad Amjad Ali, Faheem Shehzad Baloch, Rachid Lahlali

**Affiliations:** 1 Phytopathology Unit, Department of Plant and Environment Protection, Ecole Nationale d’Agriculture de Meknes, km. 10, Route Haj Kaddour, Meknès B.P. S/40, Morocco; laaslisalaheddine@gmail.com (S.-E.L.); amallhari25@gmail.com (A.H.); helhamss@enameknes.ac.ma (H.E.H.); 2 Crop Protection Unit, Agronomic and Veterinary Institute (IAV) Hassan II, Madinat Al Irfane, Rabat 10112, Morocco; 3 International Maize and Wheat Improvement Center (CIMMYT), P.O. Box 39, Emek, Ankara 06511, Türkiye; 4 School of Agriculture, The University of Jordan, Amman 11942, Jordan; 5 Biotechnology Unit, Regional Center of Agricultural Research, National Institute of Agricultural Research (INRA), Rabat 10100, Morocco; fouad.mokrini@inra.ma; 6 Laboratory of Biotechnologies and Valorization of Natural Resources, Faculty of Sciences–Agadir, Ibn Zohr University, B.P. 8106, Agadir 80000, Morocco; e.mayad@uiz.ac.ma; 7 Department of Plant Pathology, College of Plant Protection, Henan Agricultural University, Zhengzhou 450002, China; honglianli@sina.com; 8 Department of Plant Protection, Faculty of Agriculture, Bolu Abant Izzet Baysal University, Bolu 14030, Türkiye; mustafaimren@ibu.edu.tr; 9 Department of Plant Pathology, University of Agriculture, Faisalabad 38040, Pakistan; amjad.ali@uaf.edu.pk; 10 Department of Biotechnology, Faculty of Science, Mersin University, Mersin 33343, Türkiye; balochfaheem13@gmail.com; 11 Department of Genetics, Institute of Biochemistry, Sh. Rashidov Samarkand State University, Samarkand 703004, Uzbekistan

**Keywords:** *Trichoderma*, *Bacillus*, *Meloidogyne*, *Olea europaea*, biocontrol, FTIR spectroscopy

## Abstract

Root-knot nematodes (*Meloidogyne javanica* and *M. incognita*) constrain olive (*Olea europaea* L.) production and require safer alternatives to chemical nematicides. Despite the growing interest in microbial biocontrol agents, limited information is available on how application timing and microbial consortia influence nematode suppression and host biochemical responses in olive. This greenhouse study evaluated endophytic *Trichoderma* (*T. harzianum*) and *Bacillus* (*B. subtilis*), applied alone or in combination, either before or after nematode inoculation, to determine how biocontrol treatment timing influences nematode suppression, plant performance, and host biochemical responses. Across both nematode species, preventive application consistently outperformed curative application, and the combined treatment delivered the strongest overall protection. Preventive co-application reduced nematode infection, improved root and shoot growth, attenuated oxidative damage, and preserved chlorophyll more effectively than single-agent or post-inoculation treatments. Spectroscopic (Fourier Transform Infrared (FTIR)) and multivariate analyses further showed that effective treatments were associated with lower nematode-associated protein and lipid signals and stronger signatures of structural defense in root tissues. Although *M. javanica* caused greater physiological disruption than *M. incognita*, both species responded to the same general treatment pattern. These findings show that early establishment of microbial biocontrol agents is critical for durable suppression of root-knot nematodes in olive and supports preventive microbial consortia as a promising strategy for integrated nematode management.

## 1. Introduction

Root-knot nematodes (RKNs) belonging to the genus *Meloidogyne* represent one of the most destructive groups of plant-parasitic nematodes worldwide, causing severe yield losses in a wide range of perennial and annual crops, including olive (*Olea europaea* L.). These nematodes account for estimated global economic losses of approximately 80–157 billion USD annually [1,2]. Among the *Meloidogyne* species, *M. javanica* and *M. incognita* are particularly prevalent in Mediterranean olive-growing regions, where they induce characteristic root galling, impair water and nutrient uptake, and significantly compromise plant vigor and productivity [1,3,4]. In Morocco, several studies have confirmed *M. javanica* as a primary pathogen affecting olive root systems, with field surveys documenting widespread parasitism in major cultivation areas [5,6]. The rapid biological cycle of RKNs results in exponential population growth and persistent parasitic pressure, making management increasingly challenging [7].

Traditional control strategies relying on synthetic nematicides face mounting scrutiny due to environmental concerns, regulatory restrictions, and nematode resistance development, so eco-safe biological control alternatives (e.g., endophytic bacteria and fungi) are increasingly needed [8,9]. *Bacillus* species suppress nematodes through nematicidal compounds, systemic resistance, and cuticle degradation, while also managing secondary infections [10,11,12]. *Trichoderma* species act through mycoparasitism, antibiosis, competition, and nematicidal enzymes, while inducing host defense enzymes that strengthen resistance [13,14,15,16].

The application timing of biocontrol agents represents a critical yet understudied factor influencing their efficacy [17]. Preventive applications, administered before nematode inoculation, may allow microbial establishment and activation of plant defense mechanisms, potentially offering superior protection compared to curative treatments applied after infection onset [18]. Recent advances in biocontrol strategies emphasize the importance of understanding direct antagonistic interactions and indirect effects mediated through induced systemic resistance [19,20]. Endophytic colonization by beneficial fungi and bacteria can enhance biocontrol persistence and efficacy by establishing intimate associations within plant tissues, providing localized defense against nematode invasion [21].

*Trichoderma* spp. and *Bacillus* spp. have been successfully used for the enhancement of resistance in several crop plants against biotic and abiotic stresses with special reference to plant-parasitic nematodes [22,23,24,25]. However, most studies have examined these genera in isolation, in annual crops, or without explicitly testing whether application before infection provides a measurable advantage over treatment after nematode establishment. For perennial hosts such as olive, where root colonization, defense priming, and cumulative nematode pressure unfold over longer time scales, treatment timing may be as important as agent identity. Despite these advances, comprehensive studies evaluating the comparative efficacy of *Trichoderma* spp. and *Bacillus* spp. against different *Meloidogyne* species in olive cultivation remain limited, both individually and in combination. The differential virulence of *M. javanica* versus *M. incognita* on susceptible olive varieties and their responses to biocontrol interventions warrant detailed investigation. This study addresses these knowledge gaps by evaluating the biocontrol potential of endophytic fungi (*Trichoderma* spp.) and bacteria (*Bacillus* spp.) against *M. javanica* and *M. incognita* on a susceptible olive variety under controlled greenhouse conditions. Specifically, the effects of preventive (before nematode inoculation) versus curative (after inoculation) application timings on nematode population parameters, plant growth responses, biochemical profiles, and distinct molecular signatures were assessed using advanced spectroscopic techniques. The novelty of this work lies not simply in using *Trichoderma* or *Bacillus* as biocontrol agents, but in integrating agent type, application timing, and nematode species within a single olive pathosystem while also connecting classical infection measurements with biochemical and spectroscopy-based indicators of host response. In that sense, it is hypothesized that preventive application of *Trichoderma* and *Bacillus*, particularly in combination, would suppress *Meloidogyne* infection more effectively than curative application. Mechanistically, preventive application is expected to favor successful root and rhizosphere establishment of beneficial microbes before nematode penetration, thereby increasing the likelihood of direct antagonism against infective juveniles and earlier activation of induced resistance.

## 2. Materials and Methods

### 2.1. Plant Material and Growth Conditions

Root cuttings of *O. europaea* L. cv. Arbequina, a cultivar known to be susceptible to RKNs, were obtained through a licensed commercial nursery in the Meknes region of Morocco. The greenhouse experiment was conducted under controlled environmental conditions: temperature of 25 ± 2 °C, relative humidity of 60–70%, and a light/dark photoperiod of 14:10 h. A sterile substrate mixture of sandy loam soil (C:N ratio of 12:1), sand, and composted organic matter (2:1:1) was put into plastic pots with a 3 kg capacity (20 cm diameter × 25 cm height). Prior to autoclaving, the bulk soil was assessed for the presence of indigenous plant-parasitic nematodes and nematode-antagonistic microorganisms using a 200 cm^3^ subsample and standard plating on PDA and NA media. The substrate was autoclaved at 121 °C for 90 min on two consecutive days to eliminate pre-existing nematode populations and soil-borne pathogens.

### 2.2. Nematode Inoculum Preparation

Two species of root-knot nematodes, *Meloidogyne javanica* (Treub) Chitwood and *M. incognita* (Kofoid and White) Chitwood, were collected in their pure forms from crop fields naturally infested in the Souss-Massa region. These populations were subsequently cultured on tomato plants (*Solanum lycopersicum* cv. Roma, Italy), which are known to be susceptible under greenhouse settings. Verification of species identity was accomplished by examining perineal patterns and conducting molecular analysis with PCR primers specific to each species (forward (C2 F3): 5′–GGT CAA TGT TCA GAA ATT TGT GG–3′ and reverse (1108): 5′–TAC CCT CAA TTT CAA CTT TCC–3′), followed by sequencing and comparison with validated reference sequences. Species identity was further corroborated by the characteristic amplicon size differences observed between the two species upon agarose gel electrophoresis [26]. To prepare the inoculum, tomato roots bearing abundant mature egg masses were harvested 60 days after inoculation. These roots were thoroughly washed with tap water before being sectioned into 2–3 cm pieces. Extraction of eggs and second-stage juveniles (J2) was performed from root sections using a modified Baermann funnel method [27] in conjunction with a 3-min treatment using 0.5% sodium hypochlorite to dissolve the gelatinous matrix surrounding the eggs [28]. The resulting nematode suspension was rinsed with sterile distilled water and filtered through a series of nested sieves (500 μm, 150 μm, and 25 μm) to harvest newly emerged J2 after a 3-day incubation period [29]. Only freshly hatched J2s were used for inoculation, and the maximum interval between J2 collection and plant inoculation did not exceed 6 h, during which juveniles were maintained at 4 °C in sterile distilled water to preserve motility and infectivity. Before inoculation, J2 viability was confirmed by motility assessment under a stereomicroscope (Olympus LS, Tokyo, Japan), and only suspensions exhibiting ≥90% active motility were considered acceptable for use. The nematode concentration was quantified under a stereomicroscope using a counting chamber, and the suspension was diluted to achieve the target inoculum density.

### 2.3. Biocontrol Agent Preparation

Pure cultures of *Trichoderma harzianum* (isolate T1, GenBank accession number: PQ240703.1) and *Bacillus subtilis* (isolate MK3, GenBank accession number: OM791710.1), both isolated from organic/rhizosphere soils in the Fes-Meknes region and identified by ITS region sequencing and 16 S rRNA gene sequencing, respectively, were obtained from the laboratory culture collection of the Ecole Nationale d’Agriculture de Meknes (ENAM) and maintained on potato dextrose agar (PDA) and nutrient agar (NA), respectively. These specific isolates have been previously used for biocontrol assays and have confirmed their effectiveness against different plant pathogens. Actively growing *Trichoderma* spp. cultures were mass-multiplicated on sterilized wheat bran substrate under controlled conditions (25 ± 2 °C, 70% relative humidity) for 7–10 days until sporulation reached 1 × 10^8^ CFU g^−1^ (stock substrate density). To prepare the application suspension, the resulting spore suspension was then diluted to a final concentration of 1 × 10^6^ CFU mL^−1^ (final application suspensions), verified by hemocytometer count and confirmed by serial dilution plating on PDA. For *B. subtilis*, bacterial cells were grown in nutrient broth at 30 °C with continuous shaking (150 rpm) for 48 h to achieve a cell density of 1 × 10^9^ CFU mL^−1^. For greenhouse application, biocontrol agents were suspended in sterile distilled water to achieve final concentrations of 1 × 10^6^ CFU mL^−1^ for *T. harzianum* and 1 × 10^7^ CFU mL^−1^ for *B. subtilis*. Equal volumes of both suspensions were mixed immediately before application for combined treatments. The viability of the biocontrol agents was confirmed by serial dilution plating on selective media (PDA for *T. harzianum* and NA for *B. subtilis*) 24 h before each application.

### 2.4. Experimental Design and Treatments

The experiment was arranged in a completely randomized design (CRD) using a factorial core of two nematode species (*M. javanica* and *M. incognita*) and inoculated biocontrol treatments, with the negative control included as a common non-inoculated reference; each treatment had five replicates, totaling 80 experimental units. The experiment comprised eight treatments: negative control (no nematodes, no biocontrol agents; NC), positive control (nematodes only; PC), *Trichoderma* spp. applied 7 days before nematode inoculation (T−BI) or 7 days after inoculation (T−AI), *Bacillus* spp. applied 7 days before inoculation (B−BI) or 7 days after inoculation (B−AI), and the *Trichoderma* + *Bacillus* combination applied 7 days before (T+B–BI) or 7 days after inoculation (T+B−AI); for “before” treatments, agents were applied as a 50 mL soil drench around each plant’s root zone followed by a 7-day establishment period, whereas for “after” treatments nematodes were inoculated first and biocontrol agents were applied 7 days later. Nematode inoculation was performed by making four equidistant holes (3 cm deep) around the stem and depositing 1 mL aliquots of J2 suspension into each hole (1500 J2 per plant total), after which holes were immediately covered to minimize desiccation. Plants were irrigated as needed and fertilized biweekly with balanced NPK (20:20:20) at 2 g/L, and the entire experiment was independently repeated three times.

### 2.5. Nematode Population Assessment

At 9 months post-inoculation, the plants were carefully removed from the pots, and the root systems were gently washed with tap water to remove the adhering soil. Root galling severity was assessed using a 0–5 scale gall index (GI) where 0 = no galls, 1 = 1–20% of root system galls, 2 = 21–40% galled, 3 = 41–60% galled, 4 = 61–80% galled, and 5 = 81–100% of root system galls [30]. The number of egg masses per root system was quantified after staining the roots with 0.05% Phloxine B solution for 15 min. For nematode population assessment, soil from each pot was thoroughly mixed, and a 200 cm^3^ subsample was processed using the modified Baermann funnel tray method for 48 h at 25 °C to extract motile J2s. The root systems were weighed, chopped into 1–2 cm pieces, and processed using 0.5% sodium hypochlorite for egg extraction. The final nematode population (Pf) was calculated as the sum of J2 extracted from soil and eggs extracted from roots per pot. RF was calculated as RF = Pf/Pi, where Pi represents the initial inoculum density (1500 J2s) [31]. Based on RF values, the cultivars were classified as: resistant (RF < 1), moderately resistant (RF = 1–2), moderately susceptible (RF = 2–5), susceptible (RF = 5–10), or highly susceptible (RF > 10) [32].

### 2.6. Plant Growth Parameters

Plant growth was assessed by measuring the root fresh weight (RFW), root dry weight (RDW), root length (RL), root diameter (RD), shoot fresh weight (SFW), shoot dry weight (SDW), and shoot length (SL). Dry weights were determined after oven drying at 70 °C for 48 h until constant weight. The percentage change relative to the positive control was calculated using the following formula:

(1) $$Percentage\;increase\;or\;decrease=\left(\frac{Control\;Inoculated}{Control}\right)\times \;100$$

To confirm the successful establishment of the applied biocontrol agents, root colonization by *T. harzianum* and *B. subtilis* was verified at the end of the experimental period through re-isolation and selective plating. Root segments (0.5 g fresh weight) were surface-sterilized with 1% sodium hypochlorite for 2 min, rinsed three times with sterile distilled water, and plated onto *Trichoderma* selective media (TSM) and Luria–Bertani (LB) agar for fungal and bacterial recovery, respectively.

### 2.7. Biochemical Analyses

To link biocontrol efficacy with host physiology, multiple biochemical analyses were conducted. Fresh root samples (0.5 g) were collected from the feeder roots of each plant at harvest for the determination of proline content, peroxidase (POD), polyphenol oxidase (PPO), catalase (CAT), total flavonoid content (FLA), and malondialdehyde (MDA). Fresh leaf samples (0.5 g) were collected from the third fully expanded leaf from the apex for the determination of total chlorophyll content. All samples were immediately frozen in liquid nitrogen and stored at −80 °C until biochemical analysis. For proline content determination, leaf tissue was homogenized in 3% sulfosalicylic acid, and the extract was reacted with acid ninhydrin reagent at 100 °C for 1 h. The chromophore was extracted with toluene, and absorbance was measured at 520 nm using a UV–Vis spectrophotometer, with proline concentration expressed as μmol proline g^−1^ fresh weight using a standard curve [33]. Peroxidase (POD) activity was assayed by homogenizing 0.5 g leaf or root tissue in 50 mM phosphate buffer (pH 7.0) containing 1% polyvinylpyrrolidone. The reaction mixture contained enzyme extract, guaiacol, and hydrogen peroxide, and the absorbance increase was monitored at 470 nm for 3 min at 25 °C, with activity expressed as μmol guaiacol oxidized min^−1^ mg^−1^ protein [34]. Polyphenol oxidase (PPO) activity was determined using catechol as substrate in 50 mM phosphate buffer (pH 6.5), with absorbance measured at 420 nm and activity expressed as μmol catechol oxidized min^−1^ mg^−1^ protein using an extinction coefficient of ε = 1.0 mM^−1^ cm^−1^ at 420 nm [35]. Total flavonoid content was quantified using the aluminum chloride colorimetric method. Briefly, root extracts in methanol were mixed with 2% AlCl_3_ solution and incubated at room temperature for 15 min, followed by absorbance measurement at 415 nm. Total flavonoid content was expressed as mg quercetin equivalents (QEs) per g fresh weight using a quercetin standard curve [36]. Catalase (CAT) activity was measured by monitoring the decrease in absorbance at 240 nm due to H_2_ O_2_ consumption. The reaction mixture contained enzyme extract and 10–50 mM H_2_ O_2_ in 50 mM phosphate buffer (pH 7.0), with activity expressed as μmol H_2_ O_2_ decomposed min^−1^ mg^−1^ protein using an extinction coefficient of ε = 39.4 mM^−1^ cm^−1^ at 240 nm [37]. Malondialdehyde (MDA) content, an indicator of lipid peroxidation and oxidative stress, was determined using the thiobarbituric acid reactive substances (TBARSs) method. Root tissue was homogenized in 0.1% trichloroacetic acid (TCA), and the supernatant was reacted with 0.5% thiobarbituric acid in 20% TCA at 95 °C for 30 min. After cooling, absorbance was measured at 532 nm and 600 nm, with MDA content calculated using an extinction coefficient of 155 mM^−1^ cm^−1^ and expressed as μmol g^−1^ fresh weight [38].

### 2.8. Fourier Transform Infrared (FTIR) Spectroscopy Analysis

Dried and finely ground root samples (approximately 2 mg) collected from feeder and galled root regions were subjected to ATR–FTIR spectroscopy to characterize nematode-induced molecular changes in cell wall composition, protein profiles, and lipid oxidation products. Additionally, dried leaf samples (approximately 2 mg) collected from the third fully expanded leaf from the apex were analyzed to assess systemic biochemical responses and treatment differences in secondary metabolite profiles. All spectral acquisitions and processing procedures were identical for both tissue types. Spectra were acquired using a SPECTRUM TWO™ PerkinElmer spectrophotometer (PerkinElmer, Springfield, IL, USA) equipped with a diamond ATR crystal accessory. Measurements were performed in the mid-infrared region (4000–600 cm^−1^) with a spectral resolution of 4 cm^−1^ and 64 scans per sample. Background spectra were collected before each sample measurement to account for atmospheric interference. The ATR crystal was thoroughly cleaned with ethanol between samples to prevent cross-contamination. Raw spectral data were processed using PerkinElmer Spectrum™ 10 software, including baseline correction, atmospheric compensation, and normalization to eliminate systematic variation. Spectral regions corresponding to functional groups of primary and secondary metabolites were identified, including hydroxyl groups (O–H stretching, 3600–3200 cm^−1^), aliphatic groups (C–H stretching, 3000–2800 cm^−1^), carbonyl groups (C=O stretching, 1750–1650 cm^−1^), Amide I and Amide II protein bands (1650 cm^−1^ and 1540 cm^−1^), aromatic rings (C=C stretching, 1650–1450 cm^−1^), and carbohydrate regions (C–O stretching, 1200–900 cm^−1^).

### 2.9. Statistical Analysis

All statistical analyses were conducted on individual plant replicates as the experimental unit, with each treatment group comprising 5 biological replicates (n = 5). Data from the 3 independent experimental runs were analyzed using a hierarchical approach to preserve the independence of each run. For each independent experiment, the mean of the 5 replicates was first calculated, and the reported grand means and standard errors were subsequently derived from these three experiment-level means (N = 3). Prior to computing grand means, a preliminary analysis was conducted to assess inter-experiment consistency using a linear mixed-effects model alongside the intraclass correlation coefficient (ICC). The latter was in the range of 0.86–0.94 for all nematode, plant, and biochemical variables, indicating a good experimental consistency.

All data were subjected to descriptive statistical analysis, including means, standard deviations, and standard errors for each treatment combination. Normality of data distribution was assessed using the Shapiro–Wilk test, and the homogeneity of variance was verified using Levene’s test prior to analysis of variance (ANOVA). Two-way ANOVA was performed to evaluate the main effects of treatment, nematode species, and their interaction on plant growth parameters, nematode reproduction indices, and biochemical variables using the Statistical Package for the Social Sciences (SPSS) version 26.0 (IBM Corp., Armonk, NY, USA). When significant differences were detected (*p* < 0.05), means were separated using Fisher’s least (LSD) or Tukey’s honest significant difference (HSD) tests at α = 0.05. Non-parametric analyses (Kruskal–Wallis, Mann–Whitney U, and Dunn’s test with Bonferroni correction) were applied exclusively to gall index data because these variables did not satisfy normality assumptions. Multivariate analysis of variance (MANOVA) was used only to assess the combined multivariate response of plant growth and biochemical parameters across treatments.

To unveil the association between nematode density and plant growth recovery, a nonlinear regression analysis was performed using a power decay model to quantify the relationship between nematode infection pressure and plant growth responses under different biocontrol treatments. The regression analysis employed a three-parameter power decay function of the following form:

(2) $$y\;=\;a\;-\;{\mathrm{bx}}^{c}$$

where *y* represents the percentage growth increase, *x* represents nematode load (eggs and juveniles per mL soil), and *a*, *b*, and *c* are fitted parameters. The goodness of fit was evaluated using the coefficient of determination (R^2^).

A treatment–response association network was constructed to visualize the multidimensional association between biocontrol treatment and the parameters of plant and nematode biochemical responses. The weighted average of each treatment was calculated as follows:

(3) $$Score=0.4\times \left(Nematode\;Control\%\right)+0.4\times \left(Plant\;Growth\%\right)\;+0.2\times \left(Stress\;Reduction\;\%\right)$$

The preprocessed FTIR spectral data underwent linear discriminant analysis (LDA) to obtain multivariate classification of biocontrol treatments and determine the discrimination prowess between treatment groups. The analysis in both *M. javanica* and *M. incognita* species was done separately, and the analysis of the two species was done again with the incorporation of the two species. Cross-validation was done with leave-one-out cross-validation (LOOCV) to evaluate model robustness and classification accuracy. LDA and LOOCV were applied exclusively to FTIR datasets to evaluate the ability of spectral fingerprints to discriminate among treatment groups.

All statistical analyses and visualizations were performed using Python 3.14.2 with the following libraries: NumPy (version 1.24.3) for numerical operations, pandas (version 2.0.3) for data manipulation, scikit-learn (version 1.3.0) for LDA modeling and cross-validation, matplotlib (version 3.7.2) for plotting, and seaborn (version 0.12.2) for enhanced statistical graphics, implemented in PyCharm (version 2025.3; JetBrains s.r.o; Amsterdam, The Netherlands) integrated development environment (IDE).

## 3. Results

### 3.1. Effect of Biocontrol Treatments on Nematode Parameters

The evaluation of biocontrol treatments revealed significant reductions in nematode infection parameters across all treatment groups compared with the positive control, with variation depending on the biocontrol agent used, the timing of application, and the nematode species (Table 1; Figure S1). Two-way ANOVA demonstrated highly significant overall effects on gall formation, with nematode species having the largest effect (F_1,56_ = 124.56, *p* < 0.001), followed by treatment (F_6,56_ = 87.42, *p* < 0.001), while the interaction effect was smaller but still significant (F_6,56_ = 12.38, *p* < 0.001).

Similarly, egg production was strongly affected by nematode species (largest effect; F_1,56_ = 156.89, *p* < 0.001) and by treatment (F_6,56_ = 93.17, *p* < 0.001), while the treatment × species interaction was smaller but still significant (smallest effect; F_6,56_ = 15.24, *p* < 0.001). Reproduction factor showed the same pattern, with species explaining the greatest share of variation (F_1,56_ = 132.47, *p* < 0.001), treatment also highly significant (F_6,56_ = 78.95, *p* < 0.001), and the interaction remaining significant but comparatively smaller (F_6,56_ = 10.83, *p* < 0.001). Consistent with these overall tests, all biocontrol treatments reduced egg production and reproduction factor relative to the positive control (*p* < 0.05), with larger reductions generally observed under pre-inoculation than post-inoculation application. The combined pre-inoculation application (T+B–BI) demonstrated the most effective suppression across all infection parameters for both nematode species (Table 1). Against *M. javanica*, T+B–BI reduced gall numbers, egg production, and the reproduction factor by approximately 70%, 68%, and 65%, respectively (RF = 4.40 ± 0.56; F_6,28_ = 45.29, *p* < 0.001), with even greater suppression recorded against *M. incognita* (reductions of 69%, 66%, and 71%; RF = 2.76 ± 0.26; F_6,28_ = 68.43, *p* < 0.001). Among single-agent treatments, *Trichoderma* outperformed *Bacillus* (F_1,28_ = 18.47, *p* < 0.001).

**Figure 1:**
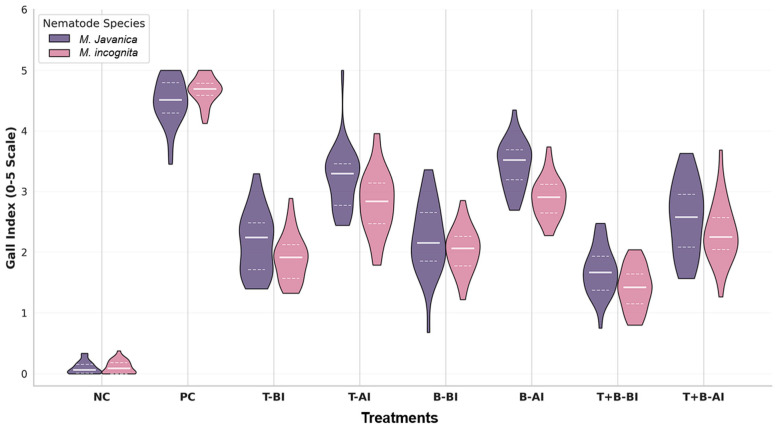
Distribution of gall indices in susceptible olive variety inoculated with different biocontrol treatments under *Meloidogyne javanica* and *M. incognita* infection. Violin plots display the frequency distribution and median values of galling severity on a 1–6 scale, with wider sections indicating higher data density. Purple and pink/red colors represent *M. javanica* and *M. incognita*, respectively. NC = negative control (without nematodes); PC = positive control (nematodes only); T–BI = *Trichoderma* applied before nematode inoculation; T–AI = *Trichoderma* applied after nematode inoculation; B–BI = *Bacillus* applied before nematode inoculation; B–AI = *Bacillus* applied after nematode inoculation; T+B–BI = *Trichoderma* + *Bacillus* applied before nematode inoculation; T+B–AI = *Trichoderma* + *Bacillus* applied after nematode inoculation. Values represent grand mean ± standard error of 3 independent experiments (N = 3) (each with 5 replicates).

The distribution of gall indices across all treatments revealed distinct patterns of disease severity, with the negative control showing no galling, whereas the positive control exhibited the highest gall indices (median = 4.75 for *M. javanica*, 4.65 for *M. incognita*) (Figure 1). All treatments applied before nematode infection resulted in lower gall formation compared to those applied after inoculation. Dunn’s post hoc pairwise comparisons with Bonferroni correction confirmed significant differences between the two application timings (*p* < 0.01), with T+B–BI showing the greatest separation from PC (Z = −8.94, *p* < 0.001).

Kruskal–Wallis analysis demonstrated highly significant differences among treatments (H_7_ = 245.38, *p* < 0.001), with nematode species exerting a significant effect on galling severity (Mann–Whitney U = 2847, *p* < 0.001). A two-way ANOVA revealed a significant treatment × species interaction (F_7,112_ = 8.67, *p* < 0.001). Violin plot analysis demonstrated that, among the biocontrol treatments applied before nematode inoculation, T+B–BI produced the most compressed distributions (F_7,112_ = 12.45, *p* < 0.001) with median values shifted toward the lower end of the galling scale for both nematode species (median = 1.60 for *M. javanica*, 1.40 for *M. incognita*). The T–BI treatment also displayed notably reduced gall indices compared to the positive control (median = 2.20 for *M. javanica*, 1.95 for *M. incognita*; U = 42, *p* < 0.001), with the frequency density concentrated at moderate severity levels (indices 2–4), whereas B–BI showed intermediate distributions between T–BI and the control (median = 2.40 for *M. javanica*, 2.15 for *M. incognita*; U = 234, *p* = 0.032 vs. T–BI). Post-inoculation treatments (T–AI, B–AI, T+B–AI) exhibited broader distributions with higher median values (T–AI: 3.35 for *M. javanica*, 2.85 for *M. incognita*; B–AI: 3.55 for *M. javanica*, 2.95 for *M. incognita*; T+B–AI: 2.60 for *M. javanica*, 2.25 for *M. incognita*) and greater overlap with the positive control distribution, particularly for *M. javanica* (Figure 1).

**Figure 2:**
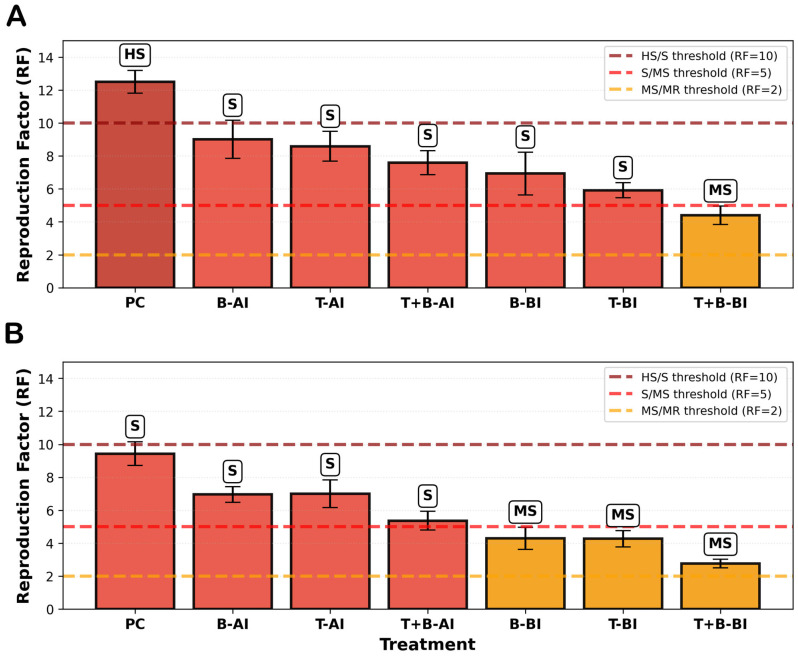
Host status transitions of susceptible olive to *Meloidogyne javanica* (**A**) and *M. incognita* (**B**) by application of biocontrol treatments. The dashed lines represent the main host status thresholds based on the reproduction factor (RF). Abbreviations stand for: HS = highly susceptible; S = susceptible; MS = moderately susceptible. PC = positive control (nematodes only); T–BI = *Trichoderma* applied before nematode inoculation; T–AI = *Trichoderma* applied after nematode inoculation; B–BI = *Bacillus* applied before nematode inoculation; B–AI = *Bacillus* applied after nematode inoculation; T+B–BI = *Trichoderma* + *Bacillus* applied before nematode inoculation; T+B–AI = *Trichoderma* + *Bacillus* applied after nematode inoculation. Values represent grand mean ± standard error of 3 independent experiments (N = 3) (each with 5 replicates).

Biocontrol treatments changed the host status of susceptible olive plants, with preventive applications showing greater reductions in the reproduction factor than post-inoculation treatments (Figure 2). While the positive control-maintained plants in a highly susceptible category (RF > 9) for both nematode species, the T+B–BI treatment successfully transitioned the host status to moderately susceptible for both *M. javanica* (RF = 4.40 ± 0.56) and *M. incognita* (RF = 2.76 ± 0.26).

Individual preventive applications of *Trichoderma* (T–BI) or *Bacillus* (B–BI) before inoculation also reduced host susceptibility to the susceptible range (RF = 4.27–6.93), though to a lesser extent than the combined treatment. In contrast, post-inoculation treatments generally maintained plants in the susceptible to highly susceptible range, with reproduction factors ranging from 5.37 to 9.01 (Figure 2).

### 3.2. Effect of Biocontrol Treatments on Plant Parameters

Nematode infection significantly impaired plant growth, with *M. javanica* causing more severe suppression than *M. incognita* across all parameters (MANOVA species effect: Wilks’ λ = 0.234, F_7,57_ = 26.73, *p* < 0.001; Table 2; Figure S2). All seven growth variables responded similarly to treatment, so root fresh weight (RFW) and shoot length (SL) were reported as representative endpoints, noting that shoot parameters were consistently more sensitive to treatment than root parameters (F_1,98_ = 67.34, *p* < 0.001). Biocontrol treatments significantly improved both variables (RFW: F_6,63_ = 34.82, *p* < 0.001; SL: F_6,63_ = 56.93, *p* < 0.001), with pre-inoculation applications outperforming post-inoculation treatments overall (timing effect: F_1,42_ = 52.18, *p* < 0.001). The combined treatment T+B–BI produced the greatest gains, as against *M. javanica,* it increased SL by 67.54% (to 27.98 ± 1.98 cm) and RFW by 29.24% (to 18.74 ± 1.33 g), while against *M. incognita*, the corresponding increases were 38.82% and 12.84%, with *M. javanica*-infected plants showing greater improvements in both cases closing the gap with the NC (Table 2). Among single-agent treatments, the timing advantage was also apparent, with T–BI improving SL by 54.91% compared to 38.02% for T–AI against *M. javanica* (F_1,16_ = 28.75, *p* < 0.001).

**Figure 3:**
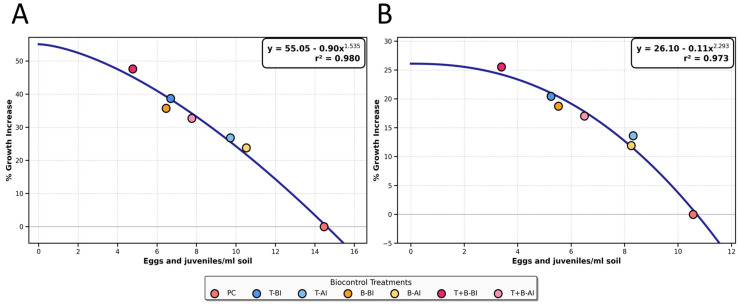
Relationship between nematode population density (eggs and second-stage juveniles per ml soil) and percentage growth increase in RKN-susceptible olive plants under biocontrol treatments. (**A**) *Meloidogyne javanica* infection. (**B**) *M. incognita* infection. Each data point represents the mean nematode density and corresponding growth improvement for each treatment. The blue lines represent the fitted regression curves. PC = positive control (nematodes only); T–BI = *Trichoderma* applied before nematode inoculation; T–AI = *Trichoderma* applied after nematode inoculation; B–BI = *Bacillus* applied before nematode inoculation; B–AI = *Bacillus* applied after nematode inoculation; T+B–BI = *Trichoderma* + *Bacillus* applied before nematode inoculation; T+B–AI = *Trichoderma* + *Bacillus* applied after nematode inoculation. The plot considered grand mean values of 3 independent experiments (N = 3) (each with 5 replicates).

**Figure 4:**
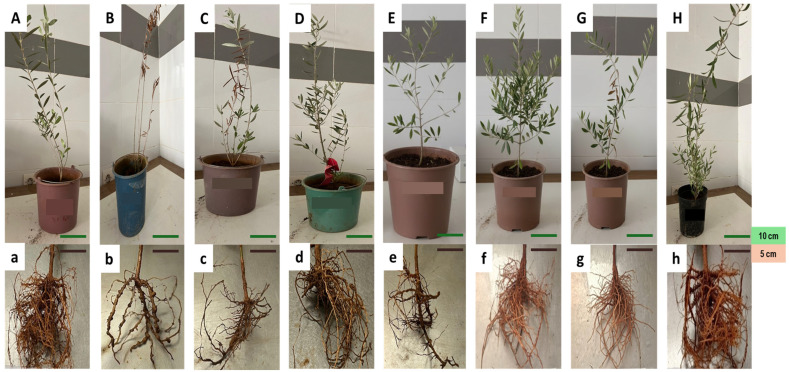
Impact of biocontrol agents applied in different scenarios against root-knot nematodes (*Meloidogyne javanica* and *M. incognita*) on aerial (upward) and rhizosphere (downward) sections of olive (Arbequina variety). (**A**,**a**) NC = negative control (without nematode/biocontrol agent); (**B**,**b**) PC = positive control (nematodes only); (**C**,**c**) T–AI = *Trichoderma* applied after nematode inoculation; (**D**,**d**) T–BI = *Trichoderma* applied before nematode inoculation; (**E**,**e**) B–AI = *Bacillus* applied after nematode inoculation; (**F**,**f**) B–BI = *Bacillus* applied before nematode inoculation; (**G**,**g**) T+B–AI = *Trichoderma* + *Bacillus* applied after nematode inoculation; (**H**,**h**) T+B–BI = *Trichoderma* + *Bacillus* applied before nematode inoculation.

Plant growth improvement declined sharply as nematode population density increased, with strong negative relationships confirmed for both *M. javanica* (r^2^ = 0.980, *p* < 0.001) and *M. incognita* (r^2^ = 0.973, *p* < 0.001) (Figure 3). The two species differed in the steepness of this response, with *M. javanica* showing a significantly greater sensitivity to nematode density than *M. incognita* (F_1,11_ = 18.94, *p* = 0.001), consistent with its higher virulence. At the extremes, T+B–BI occupied the lowest nematode densities with the highest growth improvements (~48.4% and ~25.8% for *M. javanica* and *M. incognita*, respectively; *p* < 0.001) (Figure 3). Visual assessment at 9 months corroborated these findings, with pre-inoculation treatments producing visibly healthier shoot development and less galling than post-inoculation counterparts, and T+B–BI yielding the most robust plants overall (Figure 4).

### 3.3. Effect of Biocontrol Treatments on Biochemical Parameters

Biocontrol treatments significantly reduced plant biochemical stress responses relative to the nematode-infected positive control, with application timing as the primary driver (Table 3; Figure S3). Pre-inoculation treatments (BI) achieved the greatest reductions across all stress parameters, with T+B–BI showing the most pronounced mitigation; proline declined by 59.17% and 56.65%, catalase by 78.54% and 76.17%, and MDA by 70.66% for both *M. javanica* and *M. incognita*, respectively, relative to the positive control (BI vs. AI contrast: F_1,49_ = 145.67, *p* < 0.001). In contrast, post-inoculation treatments (AI) were the least effective at reducing stress markers, with B–AI showing the smallest reductions across all parameters, proline declining by only 20.25% and 13.49%, CAT by 25.61% and 15.80%, and MDA by 21.50% and 22.63% for *M. javanica* and *M. incognita*, respectively (Table 3). MDA, a key indicator of lipid peroxidation, responded most strongly to treatment timing (F_6,70_ = 156.78, *p* < 0.001), with T+B–BI maintaining the lowest values (7.49 ± 0.52 and 7.96 ± 0.59 µmol/g FW), substantially below the PC levels of 25.53 and 27.13 µmol/g FW. Chlorophyll content, which was suppressed by nematode infection in the positive control, recovered across all biocontrol treatments, with the greatest increases observed under T+B–BI (+50.68% and +58.11% vs. PC for *M. javanica* and *M. incognita*, respectively). Flavonoid content declined in all biocontrol treatments relative to the nematode-stressed positive control (F_6,70_ = 67.89, *p* < 0.001), with the smallest reductions under AI applications. (Table 3).

### 3.4. Integrated Network Analysis of Biocontrol Treatment Effects on Susceptible Olive Infected with RKNs

**Figure 5:**
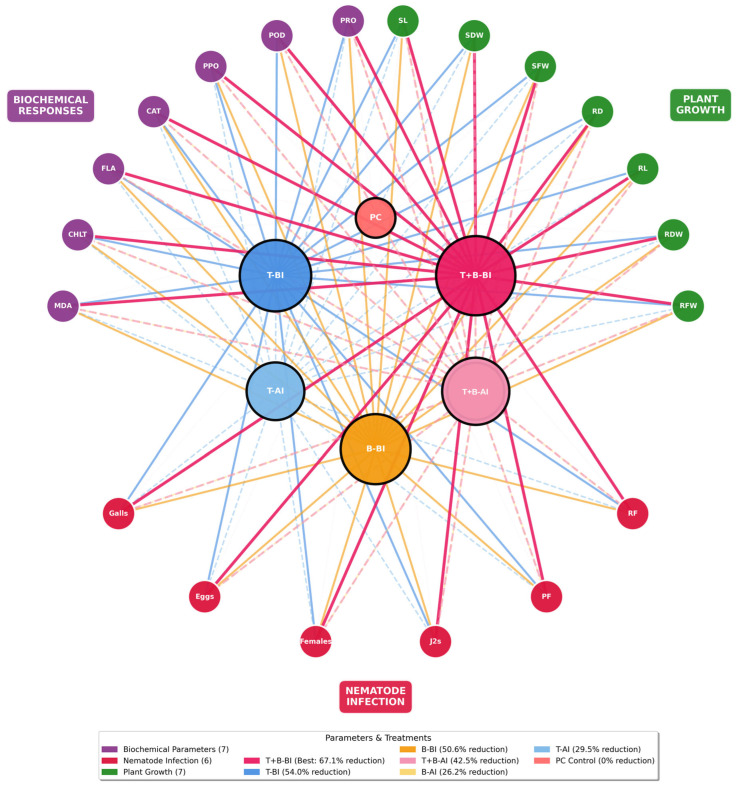
Integrated network diagram illustrates the relationships between biocontrol treatments and system-wide parameters in olive plants infected with root-knot nematodes. The network comprises seven treatment nodes (center circle) connected to 20 parameter nodes (outer ring) grouped into three functional categories: biochemical responses (violet, left sector: PRO, POD, PPO, CAT, FLA, CHLT, MDA), nematode infection indicators (red, bottom sector: galls, eggs, females, J2s, PF, RF), and plant growth metrics (green, right sector: RFW, RDW, RL, RD, SFW, SDW, SL). Treatment nodes are color-coded and sized according to effectiveness, with line styles indicating performance tiers: solid, dashed, and dotted lines represent top, moderate, and low performers, respectively. PC = positive control (nematodes only); T–BI = *Trichoderma* applied before nematode inoculation; T–AI = *Trichoderma* applied after nematode inoculation; B–BI = *Bacillus* applied before nematode inoculation; B–AI = *Bacillus* applied after nematode inoculation; T+B–BI = *Trichoderma* + *Bacillus* applied before nematode inoculation; T+B–AI = *Trichoderma* + *Bacillus* applied after nematode inoculation. The plot considered grand mean values of 3 independent experiments (N = 3) (each with 5 replicates).

The treatment–response association network integrated all 20 quantified parameters across three functional clusters: plant growth, nematode infection, and biochemical stress, and clearly distinguished treatment strategies by the strength and pattern of their connections (Figure 5). T+B–BI emerged as the most influential treatment node, showing the strongest and most consistent connections across all three clusters simultaneously (F_6,42_ = 56.78 and 89.45, respectively, *p* < 0.001). Pre-inoculation treatments as a group generated consistently stronger connections to beneficial outcomes than post-inoculation treatments (timing effect: F_1,138_ = 187.34, *p* < 0.001), while post-inoculation treatments, particularly B–AI, showed the thickest edges toward nematode infection and oxidative stress parameters. Nematode reduction and network centrality were strongly correlated across treatments (r = 0.967, *p* < 0.001). Notably, the network structure was consistent across both *M. javanica* and *M. incognita* infection scenarios (R = 0.067, *p* = 0.234) (Figure 5).

### 3.5. FTIR Spectroscopic Analysis of RKN Biocontrol Efficacy

**Figure 6:**
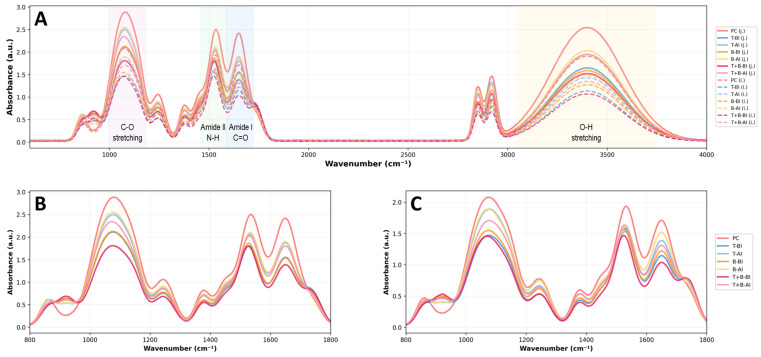
Average FTIR absorption spectra of olive root tissues following biocontrol treatment against root-knot nematode infection. (**A**) Complete spectral profile (4000–800 cm^−1^) showing characteristic vibrational bands for both *Meloidogyne javanica* (solid lines) and *M. incognita* (dashed lines) across seven treatments. (**B**) Expanded fingerprint region (1800–800 cm^−1^) for *M. javanica* showing enhanced carbohydrate and protein signature resolution. (**C**) Corresponding fingerprint region for *M. incognita* demonstrating species-specific biochemical responses. PC = positive control (nematodes only); T–BI = *Trichoderma* applied before nematode inoculation; T–AI = *Trichoderma* applied after nematode inoculation; B–BI = *Bacillus* applied before nematode inoculation; B–AI = *Bacillus* applied after nematode inoculation; T+B–BI = *Trichoderma* + *Bacillus* applied before nematode inoculation; T+B–AI = *Trichoderma* + *Bacillus* applied after nematode inoculation. The plots considered grand mean values of 3 independent experiments (N = 3) (each with 5 replicates).

FTIR spectroscopy revealed distinct biochemical signatures that differentiated olive roots treated with the biocontrol from nematode-infected controls (Figure 6). The positive control (PC) exhibited elevated absorbance intensities in the Amide I (1650 cm^−1^) and Amide II (1540 cm^−1^) regions, reflecting high nematode protein content within infected root tissues. In contrast, all biocontrol treatments demonstrated substantial reductions in these protein bands, with preventive applications (BI treatments) showing markedly greater suppression than curative applications (AI treatments). The combined *Trichoderma* + *Bacillus* treatment applied before nematode inoculation (T+B–BI) exhibited the strongest biocontrol efficacy, displaying protein band intensities approaching those of healthy, uninfected roots and representing a 50–60% reduction compared to PC for both *M. javanica* and *M. incognita*. Lipid-associated bands (2920 and 2850 cm^−1^) followed similar trends, with PC showing elevated intensities, while biocontrol treatments, particularly T+B–BI and T–BI, exhibited lower intensities (30–45%). The polysaccharide region (1000–1200 cm^−1^) revealed complex alterations in root cell wall composition, with treated samples showing distinct spectral patterns. Notably, aromatic C=C stretching bands near 1515 cm^−1^, characteristic of lignin and phenolic defense compounds, displayed enhanced intensities in BI-treated roots (15–30% increase vs. PC) (Figure 6A). Species-specific analysis demonstrated that *M. javanica* (Figure 6B) induced more severe biochemical perturbations than *M. incognita* (Figure 6C), as evidenced by 15–20% higher protein and lipid band intensities in PC samples, although both species responded similarly to biocontrol interventions. The fingerprint region (1800–800 cm^−1^) provided enhanced discrimination between treatments, with T+B–BI, T–BI, and B–BI forming a distinct spectral cluster separate from PC and AI treatments.

**Figure 7:**
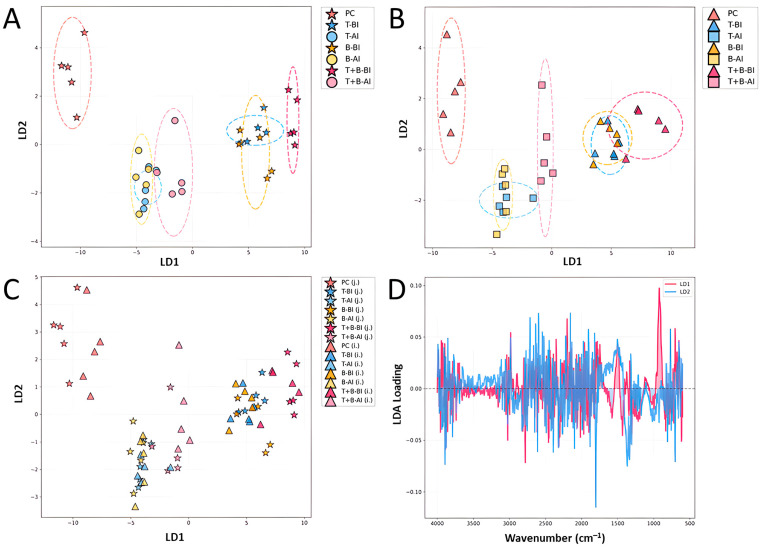
Linear discriminant analysis (LDA) of FTIR spectra discriminating the efficacy of biocontrol treatment against root-knot nematodes in olive roots. (**A**) LDA score plot for *Meloidogyne javanica* showing the separation of treatments along LD1 and LD2 axes. (**B**) Corresponding LDA score plot for *M. incognita* demonstrating species-specific clustering patterns. The dashed ellipses represent 95% confidence intervals. (**C**) Combined LDA plot integrating both nematode species, revealing consistent treatment effects across species. (**D**) LDA loading plot showing the discriminant wavenumbers contributing to treatment separation. PC = positive control (nematodes only); T–BI = *Trichoderma* applied before nematode inoculation; T–AI = *Trichoderma* applied after nematode inoculation; B–BI = *Bacillus* applied before nematode inoculation; B–AI = *Bacillus* applied after nematode inoculation; T+B–BI = *Trichoderma* + *Bacillus* applied before nematode inoculation; T+B–AI = *Trichoderma* + *Bacillus* applied after nematode inoculation. The plots considered grand mean values of 3 independent experiments (N = 3) (each with 5 replicates).

Linear discriminant analysis (LDA) of FTIR spectral data provided robust classification of biocontrol treatments with exceptional discrimination power (Figure 7). The first linear discriminant (LD1) accounted for approximately 65% of the variance and effectively separated the positive control (PC) from all biocontrol-treated samples, with the PC samples clustering in the positive LD1 region and the treated samples occupying the negative LD1 space. The second linear discriminant (LD2) explained approximately 20% of variance and distinguished preventive (BI) from curative (AI) treatments, with BI treatments exhibiting more negative LD2 values. Species-specific LDA revealed that *M. javanica* samples (Figure 7A) displayed broader dispersion in multivariate space than *M. incognita* samples (Figure 7B). Notably, the T+B–BI treatment formed the most distinct cluster, positioned furthest from PC along LD1 for both species. The 95% confidence ellipses showed minimal overlap between PC and the most effective treatments (T+B–BI, T–BI, B–BI). Combined species analysis (Figure 7C) demonstrated that treatment-induced spectral changes transcended species-specific variations, with biocontrol treatments clustering together regardless of nematode species. LDA loadings (Figure 7D) identified key discriminant wavenumbers, as LD1 exhibited prominent positive loadings at 1650 cm^−1^ (Amide I), 1540 cm^−1^ (Amide II), and 2920 cm^−1^ (lipids); LD2 showed distinctive loadings at 1515 cm^−1^ (lignin) and phenolic regions. The cross-validation analysis achieved >95% classification accuracy, with only minimal misclassification occurring between similar AI treatments. The LDA model successfully predicted treatment group membership.

**Figure 8:**
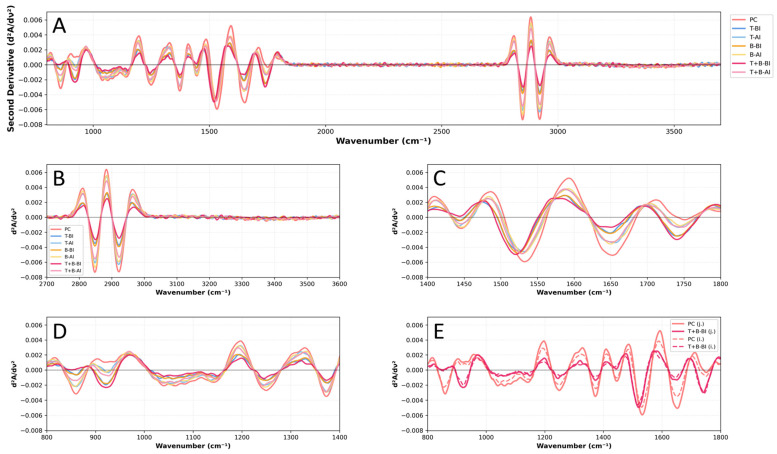
Second derivative FTIR spectra (d^2^ A/dλ^2^) of olive root tissues, revealing enhanced spectral resolution of the effects of biocontrol treatment against root-knot nematodes. (**A**) Complete second derivative spectrum (3700–800 cm^−1^) for all treatments showing sharpened peaks and enhanced overlapping band discrimination. (**B**) Lipid region (2700–3600 cm^−1^) displaying CH_2_ asymmetric (~2920 cm^−1^) and symmetric (~2850 cm^−1^) stretching vibrations of CH2. (**C**) Protein and phenolic compound region (1400–1800 cm^−1^) showing Amide I (~1650 cm^−1^), Amide II (~1540 cm^−1^), and lignin/aromatic bands (~1515 cm^−1^). (**D**) Polysaccharide fingerprint region (800–1400 cm^−1^) revealing cell wall modifications including cellulose (~1040 cm^−1^), hemicellulose (~1155 cm^−1^), and β-glucan (~920 cm^−1^) bands. (**E**) Direct comparison of PC versus T+B–BI (best treatment) across the fingerprint region for both *M. javanica* and *M. incognita.* The plot considered grand mean values of 3 independent experiments (N = 3) (each with 5 replicates).

The second derivative transformation of the FTIR spectra enhanced the resolution of the overlapping vibrational bands, revealing the molecular-level differences between the biocontrol treatments with unprecedented clarity (Figure 8). In the complete spectral profile, PC samples exhibited characteristic negative peaks at 2920, 2850, 1650, and 1540 cm^−1^ with significantly greater amplitudes than all biocontrol-treated samples (Figure 8A). The lipid region displayed sharp negative peaks at 2920 cm^−1^ (CH_2_ asymmetric stretching) and 2850 cm^−1^ (CH_2_ symmetric stretching) in the PC samples, with peak depths 2–3 times greater than those of the biocontrol treatments (Figure 8B). Preventive treatments (BI) demonstrated a 60–75% reduction in lipid peak intensities compared with PC, while curative treatments (AI) showed a 35–50% reduction. The protein and phenolic regions exhibited complex spectral patterns with distinct treatment-dependent variations (Figure 8C). PC samples showed pronounced Amide I (1650 cm^−1^) and Amide II (1540 cm^−1^) peaks, whereas BI-treated samples, particularly T+B–BI, exhibited substantially diminished protein peaks and enhanced lignin signals near 1515 cm^−1^. The second derivative analysis further resolved a shoulder peak at approximately 1600 cm^−1^, attributable to aromatic ring vibrations in phenolic defense compounds and markedly enhanced in BI treatments (intensity increase of 40–60% vs. PC). The polysaccharide fingerprint region displayed complex modifications in cell wall composition, with PC samples showing altered peak patterns at 1040 cm^−1^ (C–O stretching in cellulose) and 1155 cm^−1^ (C–O–C glycosidic bonds). Biocontrol-treated samples, especially T+B–BI and T–BI, exhibited spectral profiles more like those of healthy root tissue. Notably, the β-glucan region near 920 cm^−1^ showed treatment-specific variations, with BI treatments displaying enhanced signals (Figure 8D). Direct comparison of PC versus T+B–BI for both nematode species provided compelling visual evidence of biocontrol efficacy, with significant peak inversions across all regions of the major functional groups. *M. javanica* samples consistently exhibited larger spectral differences between PC and T+B–BI compared with *M. incognita* (Figure 8E).

Quantitative analysis of FTIR peak intensities revealed substantial molecular-level differences between preventive (BI) and curative (AI) biocontrol treatments. Preventive applications demonstrated markedly superior efficacy across all nematode-associated biomarkers compared with curative treatments. Protein-related bands, particularly Amide I (1650 cm^−1^) and Amide II (1540 cm^−1^), exhibited dramatic reductions in BI-treated samples, with intensity decreases ranging from 28.7% to 42.7% and 18.4% to 28.5%, respectively, relative to infected controls (Table 4). In contrast, AI treatments showed more modest protein reductions of 20.0–33.8% (Amide I) and 19.9–26.5% (Amide II), with 1.3–1.5–fold lower efficacy in suppressing nematode populations (Table 5). The combined T+B–BI treatment consistently produced the greatest protein reductions across both nematode species, with *M. javanica* showing a 42.7% decrease in Amide I compared to the 39.3% decrease in *M. incognita*. Lipid biomarkers (2920 and 2850 cm^−1^) followed similar trends, with BI treatments achieving reductions of 25.4–37.8% and 26.7–31.8%, respectively, compared to 15.2–26.9% and 15.8–25.8% for AI applications. Notably, the O–H/N–H stretching region (3400 cm^−1^), which encompasses both protein and hydroxyl contributions, displayed an intensity reduction of 33.2–43.9% in BI treatments versus 30.1–37.1% in AI treatments. Conversely, plant defense-associated bands revealed opposite trends, with enhanced signals in treated versus infected control samples. The stress marker region at 920 cm^−1^, attributed to C–H deformation in oxidative stress compounds and defense metabolites, exhibited remarkable increases of 101.9–146.5% in BI treatments compared to 49.1–101.0% in AI treatments. The aromatic C=C stretching band at 1515 cm^−1^, characteristic of lignin and phenolic compounds, showed complex patterns with reductions observed in both treatment strategies. Polysaccharide bands (1040, 1105, and 1155 cm^−1^) displayed treatment-dependent alterations ranging from 13.9% to 37.7% intensity decreases, reflecting both reduced nematode-induced cell wall modifications and altered host cell wall composition in biocontrol-treated roots. The pectin-associated C=O ester band (1740 cm^−1^) uniquely showed intensity increases (+8.0% to +11.8%) in BI treatments compared with more variable changes (−2.2% to +6.1%) in AI treatments. Species-specific analysis revealed that *M. javanica* induced more severe biochemical perturbations than *M. incognita* across all measured parameters, with intensity changes in infected controls being 15–25% greater. However, both species responded similarly to biocontrol interventions, with treatment efficacy ratios (BI:AI) remaining consistent at approximately 1.4:1 for lipid markers and 1.3:1 for protein markers. The T+B–BI treatment consistently outperformed individual biocontrol agents, achieving synergistic effects with 5–15% greater molecular changes compared to T–BI or B–BI alone.

## 4. Discussion

### 4.1. Integrated Suppression of Meloidogyne spp. in Olive by Trichoderma–Bacillus Consortia

The present work demonstrates that preventive application of a *Trichoderma*–*Bacillus* consortium consistently outperformed all single-agent and curative treatments across nematode infection, plant growth, and host biochemical response variables. The endophytic *Trichoderma* (*T. harzianum*) and *Bacillus* (*B. subtilis*), particularly when applied as a consortium before nematode inoculation (T+B–BI), can substantially reduce RKN infection in susceptible olive under greenhouse conditions. Across both *Meloidogyne* species, T+B–BI consistently produced the lowest values of nematode parameters. These suppression levels are comparable to, or higher than, those reported for single biocontrol strains in annual crops, where *T. asperellum* FbMi6 and commercial *Trichoderma* formulations such as T34 and T22 have been shown to reduce *M. incognita* egg production, egg masses, or overall nematode populations by roughly 40–70% in pot or field-related experiments [39,40].

The mechanistic basis for these outcomes involves complementary and reinforcing modes of action from both agents. *Trichoderma* spp. attack nematodes and their eggs through the production of cuticle- and eggshell-degrading enzymes (chitinases, proteases, and glucanases), nematicidal secondary metabolites (e.g., peptaibols, pyrones, and terpenoids), and direct parasitism of egg-associated microorganisms, while also colonizing roots endophytically and triggering phytohormonal-mediated defense pathways [41,42]. *Bacillus* spp. complement these effects by producing cyclic lipopeptides, such as surfactin, fengycin, and bacillomycin, volatile and diffusible nematicidal metabolites, hydrolytic enzymes targeting nematode cuticle structures, and phytohormones, including indole-3-acetic acid (AIA), that modulate root architecture and nutrient uptake [43,44].

When combined, these agents interact synergistically at multiple levels. The superiority of combined formulations over individual agents has been attributed to the synergistic modes of action exhibited by *Trichoderma* and *Bacillus*, including direct parasitism, antibiosis, competition for nutrients and space, and systemic resistance induction in the host plant [45]. Network analysis quantified this advantage, as T+B–BI achieved a significant nematode reduction, while simultaneously outperforming single agents on plant growth and stress parameters. This finding is consistent with growing evidence that multicomponent microbial inoculants, particularly those combining *Trichoderma* with plant growth-promoting *Bacillus* spp., perform more reliably and effectively than single strains under complex soil conditions due to functional complementarity and enhanced rhizosphere interactions [46,47,48]. These microorganisms can (i) jointly colonize the rhizoplane and outer cortical tissues, effectively excluding nematodes from infection courts, (ii) establish a metabolite-rich antagonistic microenvironment containing lipopeptides, VOCs, and lytic enzymes, and (iii) coprime plant defense networks, broadening the spectrum and durability of ISR/SAR-like responses [10,12,41,49,50,51]. An important aspect of these results is that both *M. javanica* and *M. incognita* responded similarly to the treatment ranking, despite clear species-specific differences in baseline virulence. *M. javanica* caused more severe growth suppression and stronger perturbations in untreated plants, yet T+B–BI significantly reduced infection parameters in both species, shifting RF values from highly susceptible (>9) to moderately susceptible categories. Comparable cross-species efficacy of *Trichoderma*-based products and plant growth-promoting *Bacillus* strains has been reported in vegetable crops, where substantial reductions in galling, egg production, or overall infection levels have been observed across different *Meloidogyne* species [41,49]. This indicates that the consortium may provide broad-spectrum suppression of RKNs in olive, a woody perennial for which biocontrol data remain relatively scarce.

### 4.2. Importance of Preventive Application and Host Status Modulation

A clear pattern emerging from the nematode and host status data is critical for application timing. All pre-inoculation (BI) treatments were markedly more effective than their AI counterparts in reducing galling, egg production, female counts, and RF. In contrast, the combined preventive treatment (T+B–BI) was more effective overall and shifted RF into the moderately susceptible class for both nematode species, whereas post-inoculation protocols generally left plants in the susceptible to highly susceptible range.

The mechanistic explanation for this timing effect lies in the biological prerequisites for effective biocontrol against sedentary endoparasites. The research results also support the idea that the colonization of early roots and the priming of the host defense system are key to the biological control of sedentary endoparasites, which find permanent feeding sites soon after penetration [41,49]. Prevention application will enable *Trichoderma* and *Bacillus* to settle in the rhizosphere and root tissues before nematode invasion, which may establish a biological barrier capable of intercepting infective second-stage juveniles (J2) that can settle and colonize the root cortex [52]. In addition, preventive biocontrol provides sufficient time for the activation of ISR pathways before nematode challenge. ISR priming is typically associated with enhanced responsiveness of jasmonic acid (JA) and salicylic acid (SA) signaling pathways, improved antioxidant capacity, and reinforcement of structural barriers such as lignified cell walls [53,54]. This distinction between defense priming and stress overactivation has been emphasized in research on plant–microbe interactions [55]. Comparison with the literature supports the practical relevance of this timing-based advantage [49]. The host status shift in olive from highly susceptible to moderately susceptible under T+B–BI is agronomically meaningful. When integrated with additional management tactics, RF thresholds near or below 3–5 are often associated with acceptable damage levels in annual crops, and microbial treatments that reduce RF into this range are considered successful components of integrated nematode management [49]. Such a shift suggests potential to slow root system decline and extend orchard lifespan for perennial olives, where cumulative nematode pressure over years is critical. These findings carry broad implications for biocontrol deployment in perennial systems, where the window for preventive treatment is well-defined by the season of root flush activity and orchard establishment, making timing-based microbial protocols practically feasible at the commercial scale.

### 4.3. Plant Growth Promotion and Its Tight Coupling to Nematode Suppression

One of the key findings in the plant growth dataset is that growth recovery was tightly coupled to nematode suppression rather than constituting an independent growth promotion effect. All biocontrol treatments improved plant growth parameters, but the preventive consortium treatment (T+B–BI) consistently produced the largest gains. These magnitudes are comparable to those reported for *Trichoderma*- or *Bacillus*-based treatments in vegetables and cereals, where growth and yield improvements of 20–40% under nematode pressure are frequently observed [48,56].

The strong negative power–law relationships observed between nematode density and percentage growth improvement indicate that nematode suppression, rather than direct growth promotion alone, primarily facilitated the yield benefits. This tight coupling is consistent with the dual functional nature of many *Trichoderma* and *Bacillus* strains, which simultaneously suppress pathogens and promote plant growth. These microbes enhance plant performance through phytohormone production, improved nutrient availability, enhanced root system architecture, and host hormonal signaling modulation [42,43,56,57]. Beyond these direct mechanisms, the application of *T. harzianum* and *B. subtilis* is increasingly recognized for its capacity to remodel the broader rhizosphere microbiome, a dimension particularly relevant in long-duration perennial systems such as the 9-month trial conducted in our study. Crucially, such microbiome remodeling may enrich functionally suppressive genera including *Lysobacter*, *Paenibacillus*, and *Streptomyces*, all of which produce nematicidal metabolites and lytic enzymes capable of degrading nematode egg masses and cuticle proteins, thereby providing an indirect but sustained layer of biocontrol activity that complements the direct antagonism of the applied strains [42,43].

Beyond root-level effects, the data point to a systemic dimension of biocontrol-mediated plant recovery. Observed differences in vegetative vigor among individual plants, including among uninoculated controls, reflect the inherent biological variability of woody perennials grown under pot conditions. Growth improvements were more pronounced in shoots than in roots, and the fact that shoot length exhibited the largest relative increases indicates that systemic physiological changes extended beyond direct root protection. Such systemic effects have been associated with improved photosynthetic efficiency, altered source–sink relationships, and enhanced metabolic activity in beneficial microbe-colonized plants [58,59,60,61]. This systemic dimension is particularly relevant for olive, a perennial crop where sustained canopy health over multiple seasons determines long-term productivity, and where root-targeted biocontrol may have carry-over benefits for shoot development beyond the treatment season.

### 4.4. Biochemical Defense Activation Versus Stress Mitigation Under Different Treatment Timings

The biochemical profiles reveal a clear gradient of stress mitigation depending on biocontrol agent and application timing, all assessed relative to the nematode-infected positive control, which represented the peak stress state in this study. Nematode infection alone elevated proline, POD, PPO, CAT, and MDA substantially above levels in healthy plants, confirming the well-established oxidative and osmotic stress signature of RKN parasitism in susceptible hosts.

All biocontrol treatments reduced these elevated stress markers relative to the positive control, but the magnitude of reduction differed markedly with application timing. Pre-inoculation treatments, particularly T+B–BI, achieved the greatest mitigation: proline declined by approximately 57–59%, catalase by 76–79%, and MDA by nearly 71% for both *M. javanica* and *M. incognita*. This pattern reflects a primed physiological state, where the prior establishment of *Trichoderma* and *Bacillus* in the rhizosphere allows the plant to mount a regulated and proportionate defense response, avoiding the runaway oxidative cascade that nematode infection alone provokes. Such priming phenomena are well-documented for both agents, which enhance readiness of defense pathways while maintaining metabolic homeostasis [42,43,54,62].

Post-inoculation treatments (AI), and particularly B–AI, were the least effective at reducing the nematode-induced stress burden, with proline declining by only 13–20%, catalase by 16–26%, and MDA by 21–23% relative to the positive control. This attenuated mitigation likely reflects the limited capacity of biocontrol agents to reprogram an already established stress response: by the time agents were applied, nematode colonization had triggered a sustained oxidative and osmotic imbalance that proved difficult to fully reverse. Similar observations of suboptimal biochemical recovery under curative biocontrol application have been reported in crops treated with *Trichoderma* or antagonistic bacteria against *Meloidogyne* spp. following infection onset [39,41,49,63].

Flavonoid content declined in all biocontrol treatments relative to the nematode-stressed positive control, with the smallest reductions under AI applications. A more mechanistically complete explanation lies in the metabolic architecture of the phenylpropanoid pathway itself: under induced systemic resistance (ISR) primed by *T. harzianum* and *B. subtilis*, soluble flavonoid intermediates may be actively redirected toward structural lignin biosynthesis rather than accumulating as free phenolic compounds in the soluble fraction [64,65]. This metabolic channeling would simultaneously account for two observations reported here, the lower soluble flavonoid pools in BCA-treated roots and the enhanced spectral absorbance at 1515 cm^−1^, a band characteristic of aromatic lignin C=C stretching, since both phenomena are downstream consequences of the same phenylpropanoid flux reorientation toward cell wall fortification. This distinction mirrors findings in other plant–microbe systems where *Trichoderma* and *Bacillus* spp. modulate SA, JA, and ethylene signaling pathways to induce ISR/SAR-like states that strengthen antioxidant capacity and phenolic reinforcement of cell walls without driving the plant into chronic stress [66,67]. Collectively, the biochemical data confirm that the benefit of preventive application extends beyond pathogen suppression to the preservation of host metabolic homeostasis, a distinction with practical implications for biocontrol product design and application timing in commercial olive management.

### 4.5. Fourier Transform Infrared Spectroscopy and Chemometric Classification as Non-Destructive Indicators of Biocontrol Efficacy

FTIR spectroscopy provided in-depth confirmation of nematode suppression and plant defense activation observed through classical nematological and biochemical assessments. In infected positive control roots, strong absorbance at 1650 cm^−1^ (Amide I) and 1540 cm^−1^ (Amide II) indicated high protein content associated with nematode biomass and metabolic activity within root tissues [68,69,70]. Intense lipid bands at 2920 and 2850 cm^−1^, corresponding to CH_2_ stretching vibrations in membrane lipids, may further reflect active nematode development and reproduction [71,72].

All biocontrol treatments reduced these protein and lipid signals, but preventive applications, particularly the T+B–BI treatment, produced the largest decreases. These reductions were consistent with the magnitude of nematode suppression measured through nematode parameters, supporting the use of protein and lipid bands as quantitative molecular proxies for nematode load inside roots [18,73].

In contrast to nematode-associated bands, plant defense-related spectral regions increased under effective biocontrol. The aromatic C=C stretching band near 1515 cm^−1^, largely attributed to lignin and phenolic compounds, increased by 15–30% in BI treatments, especially under T+B–BI. This pattern is indicative of enhanced lignification and phenolic deposition in root tissues, processes known to strengthen structural barriers against nematode penetration and feeding site establishment [74,75,76]. These findings align with the activation of phenylpropanoid metabolism commonly reported in *Trichoderma*- and *Bacillus*-induced systemic resistance [77,78,79,80]. Further alterations were observed in the region of polysaccharide fingerprint (1000–1200 cm^−1^) that may be indicative of restructuring associated with gall formation and giant cell development [81,82].

The diagnostic potential of the FTIR spectral fingerprint was also demonstrated by chemometric analysis. LDA achieved classification accuracy exceeding 95% (error rate <5%), clearly discriminating against infected controls and biocontrol-treated roots. The high dispersion of T+B–BI samples and the high distance between them and positive controls suggest that this analysis can effectively support the categorization of biocontrol outcomes in both *Meloidogyne* species. Thus, this method provides a rapid, non-destructive alternative to conventional nematode counting and may be applied to evaluate novel biocontrol formulations and monitor treatment outcomes under greenhouse or field conditions [83,84,85]. From a broader perspective, the integration of FTIR spectroscopy with chemometric classification represents a methodological advance for biocontrol evaluation, offering a scalable, instrument-based quality control tool applicable to both research screening and commercial biocontrol product validation.

### 4.6. Limitations of Greenhouse Conditions and Sterilized Substrate

The necessity of using the double-autoclaved substrate, although it was critical to eradicate the pre-existing population of the nematodes and to achieve clean experimental conditions, at the same time depleted the native soil microbiome, a complicated ecological community that, in natural field soils, contributes to the regulation of nematode suppression and BCA establishment, as well as plant nutrient dynamics. The absence of competing and interacting native microbial communities could have preconditioned an artificially permissive rhizosphere environment promoting the colonization of *T. harzianum* and *B. subtilis* in excess of that possible in non-sterile field soils, and could have exaggerated the magnitude of biocontrol activity. Such constraints do not negate the results, but only outline the scope of their immediate application, and support the fact that a large-scale field validation experiment, especially over a broad range of typical Moroccan soil types and climatic regions, is necessary to be able to recommend these biocontrol methods to commercial olive production with confidence.

## 5. Conclusions

This study establishes that a preventive consortium of *Trichoderma* spp. and *Bacillus* spp. constitutes a biologically coherent and agronomically viable strategy for managing RKN-susceptible olive, integrating three functional pillars, direct nematode suppression, host defense priming, and plant growth recovery, into a single, timing-dependent intervention. The preventive application strategy (T+B–BI) was highly effective, reducing nematode infection parameters while simultaneously promoting plant growth and attenuating biochemical stress responses. Critically, the superiority of pre-inoculation timing over curative application reframes biocontrol not as a reactive treatment but as a preventive rhizosphere engineering approach, positioning microbial inoculants as a foundational component of integrated nematode management in perennial olive systems rather than a supplementary rescue option. Furthermore, modulation of the rhizosphere microbiome by these biocontrol agents likely constitutes an important indirect mechanism underlying the observed efficacy, as shifts in microbial community composition can suppress nematode establishment, enhance nutrient cycling, and reinforce plant immune priming. Beyond nematode management, the application of FTIR spectroscopy coupled with LDA chemometrics introduces a molecular fingerprinting framework capable of non-destructively quantifying biocontrol outcomes at the root level, a methodological contribution with potential utility for product screening, quality assurance, and field monitoring across diverse biocontrol systems. Future work should focus on large-scale field validation and mechanistic studies to confirm these greenhouse results and explain the biological interactions behind the observed synergy.

## Figures and Tables

**Table 1:** Effect of different biocontrol treatments on nematode infection parameters of susceptible olive infected with *Meloidogyne javanica* and *M. incognita.*

Treatment	NS	Galls/RS	Eggs/RS	PF	RF
PC	*Mj*	115.0 ± 8.3 ^a^	10,012 ± 1157 ^a^	19,101 ± 1824 ^a^	12.51 ± 0.69 ^a^
	*Mi*	80.2 ± 10.6 ^ab^	7229 ± 1072 ^ab^	14,106 ± 958 ^ab^	9.44 ± 0.71 ^ab^
T–BI	*Mj*	45.6 ± 5.9 ^e^	4584 ± 698 ^cd^	8779 ± 962 ^d^	5.92 ± 0.45 ^d^
	*Mi*	34.7 ± 3.6 ^cd^	3535 ± 488 ^d^	6296 ± 1306 ^d^	4.27 ± 0.49 ^d^
T–AI	*Mj*	75.1 ± 10.8 ^b^	6396 ± 1378 ^b^	13,275 ± 1412 ^b^	8.59 ± 0.90 ^b^
	*Mi*	53.9 ± 3.1 ^b^	5808 ± 1028 ^b^	9130 ± 621 ^c^	7.01 ± 0.84 ^b^
B–BI	*Mj*	50.6 ± 5.7 ^d^	4225 ± 831 ^cd^	9705 ± 1109 ^c^	6.93 ± 1.30 ^d^
	*Mi*	41.4 ± 3.2 ^c^	3851 ± 411 ^cd^	6836 ± 1004 ^d^	4.30 ± 0.67 ^d^
B–AI	*Mj*	76.9 ± 8.1 ^b^	6892 ± 1125 ^b^	12,224 ± 1763 ^b^	9.01 ± 1.15 ^b^
	*Mi*	56.6 ± 9.3 ^b^	5880 ± 383 ^b^	9937 ± 665 ^b^	6.96 ± 0.47 ^b^
T+B–BI	*Mj*	34.8 ± 4.3 ^e^	3173 ± 531 ^d^	6389 ± 1133 ^e^	4.40 ± 0.56 ^e^
	*Mi*	24.5 ± 3.5 ^d^	2449 ± 269 ^e^	4818 ± 480 ^e^	2.76 ± 0.26 ^e^
T+B–AI	*Mj*	65.4 ± 4.8 ^c^	4946 ± 397 ^c^	9361 ± 779 ^cd^	7.59 ± 0.73 ^c^
	*Mi*	44.8 ± 8.4 ^b^	4579 ± 960 ^c^	7536 ± 1126 ^d^	5.37 ± 0.57 ^c^

Values represent grand mean ± standard error of 3 independent experiments (N = 3) (each with 5 replicates). Different letters within each column indicate significant differences according to Fisher’s LSD test (*p* < 0.05). NS = nematode species; RS = root system; PF = final population; RF = reproduction factor. *Mj* = *Meloidogyne javanica*; *Mi* = *M. incognita*. PC = positive control (nematodes only); T–BI = *Trichoderma* applied before nematode inoculation; T–AI = *Trichoderma* applied after nematode inoculation; B–BI = *Bacillus* applied before nematode inoculation; B–AI = *Bacillus* applied after nematode inoculation; T+B–BI = *Trichoderma* + *Bacillus* applied before nematode inoculation; T+B–AI = *Trichoderma* + *Bacillus* applied after nematode inoculation. The nematode parameter measures were null for the negative control (NC).

**Table 2:** Effect of biocontrol treatments on the plant growth parameters of susceptible olive under nematode stress (*Meloidogyne javanica* and *M. incognita*).

Plant Parameters			Root				Shoot		
Treatment	Nematode Species	Inoculation Status	RFW (g)	RDW (g)	RL (cm)	RD (mm)	SFW (g)	SDW (g)	SL (cm)
NC	N/A	N/A	19.80 ± 0.84 ^a^	13.40 ± 0.92 ^a^	25.20 ± 1.02 ^a^	3.8 ± 0.3 ^a^	29.20 ± 1.84 ^a^	20.60 ± 1.36 ^a^	30.80 ± 1.66 ^a^
PC	*Mj*	Control	14.50 ± 0.80 ^b^	11.50 ± 0.90 ^b^	16.00 ± 1.11 ^c^	2.50 ± 0.16 ^c^	18.20 ± 0.84 ^c^	13.10 ± 0.61 ^c^	16.70 ± 0.71 ^c^
	*Mi*	Control	16.90 ± 1.26 ^a^	13.00 ± 0.83 ^a^	18.90 ± 1.29 ^b^	3.20 ± 0.13 ^b^	21.60 ± 1.70 ^b^	15.40 ± 1.13 ^b^	20.30 ± 0.98 ^b^
T–BI	*Mj*	Inoculated Increase (%)	17.95 ± 0.85 ^a^ 23.79	12.73 ± 0.60 ^ab^ 10.70	21.98 ± 1.15 ^ab^ 37.38	3.34 ± 0.20 ^a^ 33.60	25.35 ± 1.45 ^ab^ 39.29	17.98 ± 0.93 ^b^ 37.25	25.87 ± 1.67 ^ab^ 54.91
	*Mi*	Inoculated Increase (%)	18.64 ± 0.85 ^a^ 10.30	13.24 ± 0.68 ^a^ 1.85	22.68 ± 1.24 ^a^ 20.00	3.56 ± 0.21 ^ab^ 11.25	26.16 ± 1.87 ^a^ 21.11	18.52 ± 0.89 ^ab^ 20.26	26.60 ± 1.61 ^ab^ 31.03
T–AI	*Mj*	Inoculated Increase (%)	16.89 ± 1.08 ^a^ 16.48	12.36 ± 0.52 ^ab^ 7.48	20.14 ± 1.30 ^bc^ 25.88	3.08 ± 0.14 ^b^ 23.20	23.15 ± 0.99 ^b^ 27.20	16.48 ± 1.28 ^bc^ 25.80	23.05 ± 1.81 ^b^ 38.02
	*Mi*	Inoculated Increase (%)	18.06 ± 1.31 ^a^ 6.86	13.16 ± 0.69 ^a^ 1.23	21.42 ± 0.94 ^ab^ 13.33	3.44 ± 0.23 ^a^ 7.50	24.64 ± 1.42 ^b^ 14.07	17.48 ± 0.78 ^ab^ 13.51	24.50 ± 1.47 ^b^ 20.69
B–BI	*Mj*	Inoculated Increase (%)	17.68 ± 0.73 ^a^ 21.93	12.64 ± 0.97 ^ab^ 9.91	21.52 ± 1.08 ^ab^ 34.50	3.28 ± 0.22 ^ab^ 31.20	24.80 ± 1.30 ^ab^ 36.26	17.60 ± 1.07 ^b^ 34.35	25.16 ± 1.56 ^b^ 50.66
	*Mi*	Inoculated Increase (%)	18.50 ± 0.88 ^a^ 9.47	13.22 ± 1.04 ^a^ 1.69	22.36 ± 1.59 ^a^ 18.31	3.53 ± 0.27 ^ab^ 10.31	25.78 ± 1.95 ^ab^ 19.35	18.26 ± 1.17 ^ab^ 18.57	26.08 ± 2.00 ^ab^ 28.47
B–AI	*Mj*	Inoculated Increase (%)	16.62 ± 0.72 ^ab^ 14.62	12.26 ± 0.59 ^b^ 6.61	19.68 ± 0.82 ^b^ 23.00	3.02 ± 0.16 ^ab^ 20.80	22.60 ± 1.26 ^b^ 24.18	16.10 ± 0.82 ^b^ 22.90	22.34 ± 1.63 ^b^ 33.77
	*Mi*	Inoculated Increase (%)	17.91 ± 0.97 ^a^ 5.98	13.14 ± 0.67 ^a^ 1.08	21.10 ± 1.30 ^b^ 11.64	3.41 ± 0.16 ^ab^ 6.56	24.26 ± 1.75 ^b^ 12.31	17.22 ± 0.74 ^ab^ 11.82	23.98 ± 1.91 ^b^ 18.13
T+B–BI	*Mj*	Inoculated Increase (%)	18.74 ± 1.33 ^a^ 29.24	13.02 ± 0.62 ^ab^ 13.22	23.36 ± 0.94 ^a^ 46.00	3.54 ± 0.26 ^ab^ 41.60	27.00 ± 1.84 ^ab^ 48.35	19.10 ± 1.32 ^a^ 45.80	27.98 ± 1.98 ^a^ 67.54
	*Mi*	Inoculated Increase (%)	19.07 ± 0.82 ^a^ 12.84	13.30 ± 0.72 ^a^ 2.31	23.62 ± 1.05 ^a^ 24.97	3.65 ± 0.27 ^ab^ 14.06	27.30 ± 1.77 ^ab^ 26.39	19.30 ± 1.03 ^ab^ 25.32	28.18 ± 1.20 ^a^ 38.82
T+B–AI	*Mj*	Inoculated Increase (%)	17.41 ± 0.91 ^a^ 20.07	12.54 ± 0.66 ^a^ 9.04	21.06 ± 1.46 ^b^ 31.62	3.21 ± 0.21 ^b^ 28.40	24.25 ± 1.83 ^b^ 33.24	17.23 ± 1.01 ^b^ 31.53	24.46 ± 1.10 ^b^ 46.47
	*Mi*	Inoculated Increase (%)	18.35 ± 1.26 ^a^ 8.58	13.20 ± 0.93 ^a^ 1.54	22.05 ± 1.38 ^a^ 16.67	3.50 ± 0.25 ^ab^ 9.37	25.40 ± 1.52 ^ab^ 17.59	18.00 ± 1.10 ^a^ 16.88	25.55 ± 1.46 ^ab^ 25.86

Values represent grand mean ± standard error of 3 independent experiments (N = 3) (each with 5 replicates). Increase (%) calculated relative to the positive control (inoculated without biocontrol treatment). Different letters within each column indicate significant differences according to Fisher’s LSD test (*p* < 0.05). RFW = root fresh weight; RDW = root dry weight; RL = root length; RD = root diameter; SFW = shoot fresh weight; SDW = shoot dry weight; SL = shoot length. NS = nematode species; Mj = *Meloidogyne javanica*; Mi = *M. incognita*. NC = negative control; PC = positive control (nematodes only); T–BI = *Trichoderma* applied before nematode inoculation; T–AI = *Trichoderma* applied after nematode inoculation; B–BI = *Bacillus* applied before nematode inoculation; B–AI = *Bacillus* applied after nematode inoculation; T+B–BI = *Trichoderma* + *Bacillus* applied before nematode inoculation; T+B–AI = *Trichoderma* + *Bacillus* applied after nematode inoculation. N/A = not applicable.

**Table 3:** Biochemical responses of susceptible olive to root-knot nematode infection by *Meloidogyne javanica* and *M. incognita* under different biocontrol strategies.

Biochemical Parameters			Osmolytes	Defense-Related Enzymes			Defense-Related Secondary Metabolites	Photosynthetic Pigments	Oxidative Stress Markers
Treatment	Nematode Species	Inoculation Status	PRO (µmol/g FW)	POD (µmol.min^−1^.mg^−1^ Prot)	PPO (µmol.min^−1^.mg^−1^ Prot)	CAT (µmol H_2_ O_2_/mg Prot.min)	FLA (mg/g FW)	CHL_T_ (mg/g FW)	MDA (µmol/g FW)
NC	N/A	N/A	29.69 ± 2.25 ^d^	3.97 ± 0.65 ^c^	5.12 ± 0.56 ^c^	0.10 ± 0.01 ^f^	10.59 ± 1.06 ^c^	2.31 ± 0.19 ^a^	4.82 ± 0.42 ^d^
PC	*Mj*	Control	87.33 ± 6.99 ^a^	14.44 ± 1.73 ^a^	19.25 ± 2.12 ^a^	4.10 ± 0.33 ^a^	19.49 ± 1.75 ^a^	1.46 ± 0.15 ^efg^	25.53 ± 2.04 ^a^
	*Mi*	Control	86.29 ± 6.90 ^a^	13.55 ± 1.63 ^a^	18.39 ± 2.02 ^a^	3.86 ± 0.31 ^a^	20.27 ± 1.82 ^a^	1.48 ± 0.15 ^e^	27.13 ± 2.17 ^a^
T−BI	*Mj*	Inoculated Change (%)	45.71 ± 2.45 ^cd^ −47.66	7.45 ± 1.22 ^bc^ −48.41	10.41 ± 1.01 ^b^ −45.92	1.47 ± 0.10 ^de^ −64.15	12.44 ± 0.88 ^c^ −36.17	2.00 ± 0.17 ^ab^ +36.99	11.03 ± 1.27 ^c^ −56.80
	*Mi*	Inoculated Change (%)	46.59 ± 4.49 ^c^ −46.01	7.05 ± 0.63 ^bc^ −47.97	11.14 ± 1.11 ^b^ −39.42	1.55 ± 0.09 ^d^ −59.84	14.55 ± 1.76 ^b^ −28.22	2.07 ± 0.04 ^ab^ +39.86	12.11 ± 1.77 ^cd^ −55.36
T−AI	*Mj*	Inoculated Change (%)	62.24 ± 3.99 ^ab^ −28.73	10.52 ± 1.21 ^ab^ −27.15	14.38 ± 2.20 ^a^ −25.30	2.69 ± 0.14 ^b^ −34.39	16.13 ± 0.97 ^b^ −17.24	1.80 ± 0.08 ^b^ +23.29	17.30 ± 1.72 ^b^ −32.24
	*Mi*	Inoculated Change (%)	63.60 ± 2.60 ^ab^ −26.30	11.22 ± 0.91 ^ab^ −17.20	15.08 ± 1.17 ^a^ −18.00	2.92 ± 0.32 ^b^ −24.35	16.32 ± 1.54 ^ab^ −19.49	1.74 ± 0.15 ^bc^ +17.57	16.86 ± 0.97 ^b^ −37.85
B−BI	*Mj*	Inoculated Change (%)	53.90 ± 4.21 ^b^ −38.28	7.14 ± 0.87 ^bc^ −50.55	10.80 ± 0.92 ^b^ −43.90	1.70 ± 0.26 ^cd^ −58.54	14.63 ± 1.41 ^ab^ −24.94	1.85 ± 0.09 ^b^ +26.71	13.03 ± 1.02 ^bc^ −48.96
	*Mi*	Inoculated Change (%)	50.95 ± 2.60 ^c^ +40.95	8.33 ± 1.00 ^bc^ −38.52	11.34 ± 0.83 ^ab^ −38.34	1.77 ± 0.11 ^cd^ −54.15	14.15 ± 1.28 ^bc^ −30.19	1.97 ± 0.09 ^ab^ +33.11	13.48 ± 0.42 ^c^ −50.31
B−AI	*Mj*	Inoculated Change (%)	69.65 ± 2.57 ^a^ −20.25	11.46 ± 0.82 ^a^ −20.64	16.85 ± 1.56 ^a^ −12.47	3.05 ± 0.21 ^a^ −25.61	18.62 ± 1.55 ^a^ −4.46	1.65 ± 0.15 ^b^ +13.11	20.04 ± 1.47 ^a^ −21.50
	*Mi*	Inoculated Change (%)	74.65 ± 5.21 ^a^ −13.49	12.22 ± 1.46 ^a^ −9.82	16.59 ± 1.58 ^a^ −9.79	3.25 ± 0.27 ^a^ −15.80	17.73 ± 2.18 ^a^ −12.53	1.59 ± 0.08 ^c^ +7.43	20.99 ± 0.74 ^a^ −22.63
T+B−BI	*Mj*	Inoculated Change (%)	35.66 ± 3.49 ^cd^ −59.17	5.25 ± 0.76 ^c^ −63.64	8.58 ± 1.03 ^bc^ −55.43	0.88 ± 0.05 ^e^ −78.54	11.55 ± 0.98 ^c^ −40.74	2.20 ± 0.18 ^b^ +50.68	7.49 ± 0.52 ^cd^ −70.66
	*Mi*	Inoculated Change (%)	37.41 ± 1.87 ^cd^ −56.65	5.47 ± 0.28 ^c^ −59.63	8.40 ± 2.00 ^c^ −54.32	0.92 ± 0.07 ^e^ −76.17	12.16 ± 1.21 ^c^ −40.01	2.34 ± 0.18 ^a^ +58.11	7.96 ± 0.59 ^d^ −70.86
T+B−AI	*Mj*	Inoculated Change (%)	55.93 ± 4.48 ^b^ −35.96	8.40 ± 1.57 ^b^ −41.83	12.70 ± 1.70 ^ab^ −34.03	1.99 ± 0.20 ^c^ −51.46	13.94 ± 1.71 ^c^ −28.48	1.93 ± 0.08 ^b^ +32.19	13.95 ± 0.88 ^bc^ −45.36
	*Mi*	Inoculated Change (%)	54.79 ± 1.30 ^b^ −36.50	8.98 ± 1.03 ^b^ −33.73	12.04 ± 1.01 ^ab^ −34.53	2.12 ± 0.14 ^c^ −45.08	16.44 ± 1.46 ^bc^ −18.89	1.83 ± 0.12 ^b^ +23.65	14.58 ± 0.90 ^bc^ −46.26

Values represent grand mean ± standard error of 3 independent experiments (N = 3) (each with 5 replicates). Reduction percentage indicates growth suppression relative to non-inoculated controls. Different letters within each column indicate significant differences according to Fisher’s LSD test (*p* < 0.05). PRO = proline; POD = peroxidase; PPO = polyphenol oxidase; CAT = catalase; FLA = flavonoid content; CHL_T_ = total chlorophyll content; MDA = malondialdehyde content. *Mj*: *Meloidogyne javanica*; *Mi*: *M. incognita*. NC = negative control; PC = positive control (nematodes only); T–BI = *Trichoderma* applied before nematode inoculation; T–AI = *Trichoderma* applied after nematode inoculation; B–BI = *Bacillus* applied before nematode inoculation; B–AI = *Bacillus* applied after nematode inoculation; T+B–BI = *Trichoderma* + *Bacillus* applied before nematode inoculation; T+B–AI = *Trichoderma* + *Bacillus* applied after nematode inoculation. Change (%) indicates change relative to the positive control (PC).

**Table 4:** FTIR peak assignments and relative intensity changes (%) in olive root tissues following biocontrol treatments applied before nematode inoculation.

FTIR Parameters			Percentage Changes (Δ)					
Wavenumber (cm^−1^)	Vibration Mode	Chemical Assignment	T–BI (*Mj*)	B–BI (*Mj*)	T+B–BI (*Mj*)	T–BI (*Mi*)	B–BI (*Mi*)	T+B–BI (*Mi*)
3400	O–H, N–H stretching	Hydroxyl groups, proteins, water	−35.3%	−37.5%	−40.4%	−40.4%	−33.2%	−43.9%
2920	CH_2_ asymmetric stretching	Lipids, cell membranes	−27.5%	−26.1%	−35.4%	−28.6%	−25.4%	−37.8%
2850	CH_2_ symmetric stretching	Lipids, fatty acids	−29.8%	−27.9%	−31.8%	−25.9%	−26.7%	−31.5%
1740	C=O ester stretching	Pectins, cell wall components	+11.4%	+8.6%	+8.0%	+9.4%	+11.8%	+10.6%
1650	Amide I (C=O stretching)	Proteins, structural proteins	−35.8%	−36.4%	−42.7%	−33.0%	−28.7%	−39.3%
1540	Amide II (N–H bending)	Proteins, enzymatic proteins	−28.0%	−25.8%	−28.5%	−18.4%	−19.9%	−25.0%
1515	Aromatic C=C stretching	Lignin, phenolic compounds	−26.1%	−23.8%	−26.4%	−16.8%	−18.4%	−23.2%
1450	CH_2_ bending	Lipids, proteins	−22.1%	−18.7%	−27.6%	−22.1%	−21.8%	−31.2%
1375	CH_3_ symmetric bending	Lipids, methyl groups	−28.4%	−26.5%	−33.0%	−27.3%	−21.3%	−33.2%
1245	Amide III	Proteins, collagen–like proteins	−28.9%	−26.4%	−35.5%	−31.1%	−20.5%	−31.6%
1155	C–O–C asymmetric stretching	Cellulose, hemicellulose	−26.3%	−25.0%	−35.7%	−30.3%	−26.8%	−35.2%
1105	C–O stretching	Carbohydrates, glucose polymers	−27.1%	−26.7%	−37.7%	−29.1%	−25.7%	−31.5%
1040	C–O stretching	Polysaccharides, starch, cellulose	−25.9%	−25.5%	−36.4%	−29.6%	−25.2%	−28.9%
920	C–H deformation	Stress markers, oxidative damage	+129.3%	+131.1%	+146.5%	+101.9%	+111.7%	+124.1%
860	C–H deformation	Cellulose, β-glucans	−12.8%	−9.8%	−14.5%	−15.0%	−7.6%	−13.7%

Peak intensities are reported as normalized absorbance values (grand mean values of 3 independent experiments (N = 3), each with 5 replicates). The wavenumber positions (cm^−1^) correspond to the characteristic vibrational modes of specific functional molecular groups. Chemical assignments indicate the primary biomolecular components contributing to each absorption band. Percentage changes (Δ) are calculated as [(Infected Control)/Control] × 100, with positive values indicating increased accumulation and negative values indicating depletion of the assigned molecular components. *Mj*: *Meloidogyne javanica*; Mi: *M. incognita*.

**Table 5:** FTIR peak assignments and relative intensity changes (%) in olive root tissues following biocontrol treatments applied after nematode inoculation.

FTIR Parameters			Percentage Changes (Δ)					
Wavenumber (cm^−1^)	Vibration Mode	Chemical Assignment	T–BI (*Mj*)	B–BI (*Mj*)	T+B–BI (*Mj*)	T–BI (*Mi*)	B–BI (*Mi*)	T+B–BI (*Mi*)
3400	O–H, N–H stretching	Hydroxyl groups, proteins, water	−31.4%	−28.2%	−32.2%	−32.8%	−30.1%	−37.1%
2920	CH_2_ asymmetric stretching	Lipids, cell membranes	−15.2%	−18.1%	−28.3%	−21.4%	−18.4%	−26.9%
2850	CH_2_ symmetric stretching	Lipids, fatty acids	−17.6%	−10.5%	−23.4%	−18.8%	−15.8%	−25.8%
1740	C=O ester stretching	Pectins, cell wall components	+2.4%	–2.2%	+3.5%	+6.1%	+1.2%	+11.8%
1650	Amide I (C=O stretching)	Proteins, structural proteins	−30.5%	−29.2%	−33.8%	−27.2%	−20.0%	−31.8%
1540	Amide II (N–H bending)	Proteins, enzymatic proteins	−24.1%	−22.0%	−24.3%	−26.5%	−21.9%	−20.1%
1515	Aromatic C=C stretching	Lignin, phenolic compounds	−22.8%	−20.7%	−22.6%	−25.3%	−20.8%	−18.5%
1450	CH_2_ bending	Lipids, proteins	−18.0%	−14.2%	−21.6%	−21.1%	−16.4%	−22.5%
1375	CH_3_ symmetric bending	Lipids, methyl groups	−17.8%	−14.5%	−22.4%	−15.5%	−14.2%	−18.6%
1245	Amide III	Proteins, collagen–like proteins	−22.8%	−19.1%	−27.1%	−19.3%	−8.6%	−25.1%
1155	C–O–C asymmetric stretching	Cellulose, hemicellulose	−19.9%	−16.8%	−29.0%	−16.6%	−16.9%	−25.6%
1105	C–O stretching	Carbohydrates, glucose polymers	−19.1%	−17.1%	−28.2%	−13.9%	−14.5%	−25.5%
1040	C–O stretching	Polysaccharides, starch, cellulose	−17.4%	−15.2%	−24.8%	−15.3%	−13.9%	−26.7%
920	C–H deformation	Stress markers, oxidative damage	+69.3%	+84.2%	+101.0%	+49.1%	+64.1%	+82.6%
860	C–H deformation	Cellulose, β-glucans	−7.7%	−4.5%	−16.9%	−13.5%	−9.4%	−14.2%

Peak intensities are reported as normalized absorbance values (grand mean values of 3 independent experiments (N = 3), each with 5 replicates). The wavenumber positions (cm^−1^) correspond to the characteristic vibrational modes of specific functional molecular groups. Chemical assignments indicate the primary biomolecular components contributing to each absorption band. Percentage changes (Δ) are calculated as [(Infected Control)/Control] × 100, with positive values indicating increased accumulation and negative values indicating depletion of the assigned molecular components. *Mj*: *Meloidogyne javanica*; Mi: *M. incognita*.

## Data Availability

The datasets that support the findings of this study are contained within the article.

## Supplementary Materials

The following supporting information can be downloaded at: https://www.mdpi.com/article/10.3390/pathogens15060624/s1, Figure S1: Scatter plots of individual replicate values per experimental run for nematode infestation parameters across treatments and nematode species; Figure S2: Scatter plots of individual replicate values per experimental run for plant growth parameters under different nematode biocontrol treatments; Figure S3: Scatter plots of individual replicate values per experimental run for biochemical markers across biocontrol treatments used against root-knot nematodes.

## Abbreviations

The following abbreviations are used in this manuscript:

AI	After inoculation
AIA	Indole-3-acetic acid
ANOVA	Analysis of variance
ATR	Attenuated total reflectance
ATR–FTIR	Attenuated total reflectance Fourier Transform Infrared spectroscopy
B AI	*Bacillus* applied after inoculation
B BI	*Bacillus* applied before inoculation
BI	Before inoculation
CAT	Catalase
CFUs	Colony forming units
CRD	Completely randomized design
FLA	Total flavonoid content
FTIR	Fourier Transform Infrared spectroscopy
GI	Gall index
HSD	Tukey’s honest significant difference
ICC	Intraclass correlation coefficient
IDE	Integrated development environment
ISR	Induced systemic resistance
J2	Second-stage juveniles
JA	Jasmonic acid
LD1	First linear discriminant
LD2	Second linear discriminant
LDA	Linear discriminant analysis
LOOCV	Leave-one-out cross-validation
LSD	Fisher’s least significant difference
MANOVA	Multivariate analysis of variance
MD	Mahalanobis distance
MDA	Malondialdehyde
NA	Nutrient agar
NC	Negative control
NLSs	Nonlinear least squares
NPK	Nitrogen, phosphorus, potassium
PC	Positive control
PCR	Polymerase chain reaction
PDA	Potato dextrose agar
Pf	Final nematode population
Pi	Initial inoculum density
POD	Peroxidase
PPO	Polyphenol oxidase
QEs	Quercetin equivalents
RD	Root diameter
RDW	Root dry weight
RF	Reproduction factor
RFW	Root fresh weight
RKNs	Root-knot nematodes
RL	Root length
rpm	Revolutions per minute
SA	Salicylic acid
SAR	Systemic acquired resistance
SDW	Shoot dry weight
SFW	Shoot fresh weight
SL	Shoot length
SPSS	Statistical Package for the Social Sciences
T AI	*Trichoderma* applied after inoculation
T BI	*Trichoderma* applied before inoculation
T+B AI	*Trichoderma* + *Bacillus* applied after inoculation
T+B BI	*Trichoderma* + *Bacillus* applied before inoculation
TBARSs	Thiobarbituric acid reactive substances
TCA	Trichloroacetic acid
UV–Vis	Ultraviolet–visible
VOC	Volatile organic compound
