# Supplementary material for: Preventive Versus Curative Application of Endophytic Trichoderma harzianum and Bacillus subtilis for Biological Control of Meloidogyne javanica and M. incognita in Olive Under Greenhouse Conditions
Source: Pathogens. 2026 Jun 11;15(6):624. doi: 10.3390/pathogens15060624 (PMC13304642; doi:10.3390/pathogens15060624)
Supplement: Supplementary file 1 [file pathogens-15-00624-s001.zip › pathogens-4286432R3 Supplementary Materials.pdf]

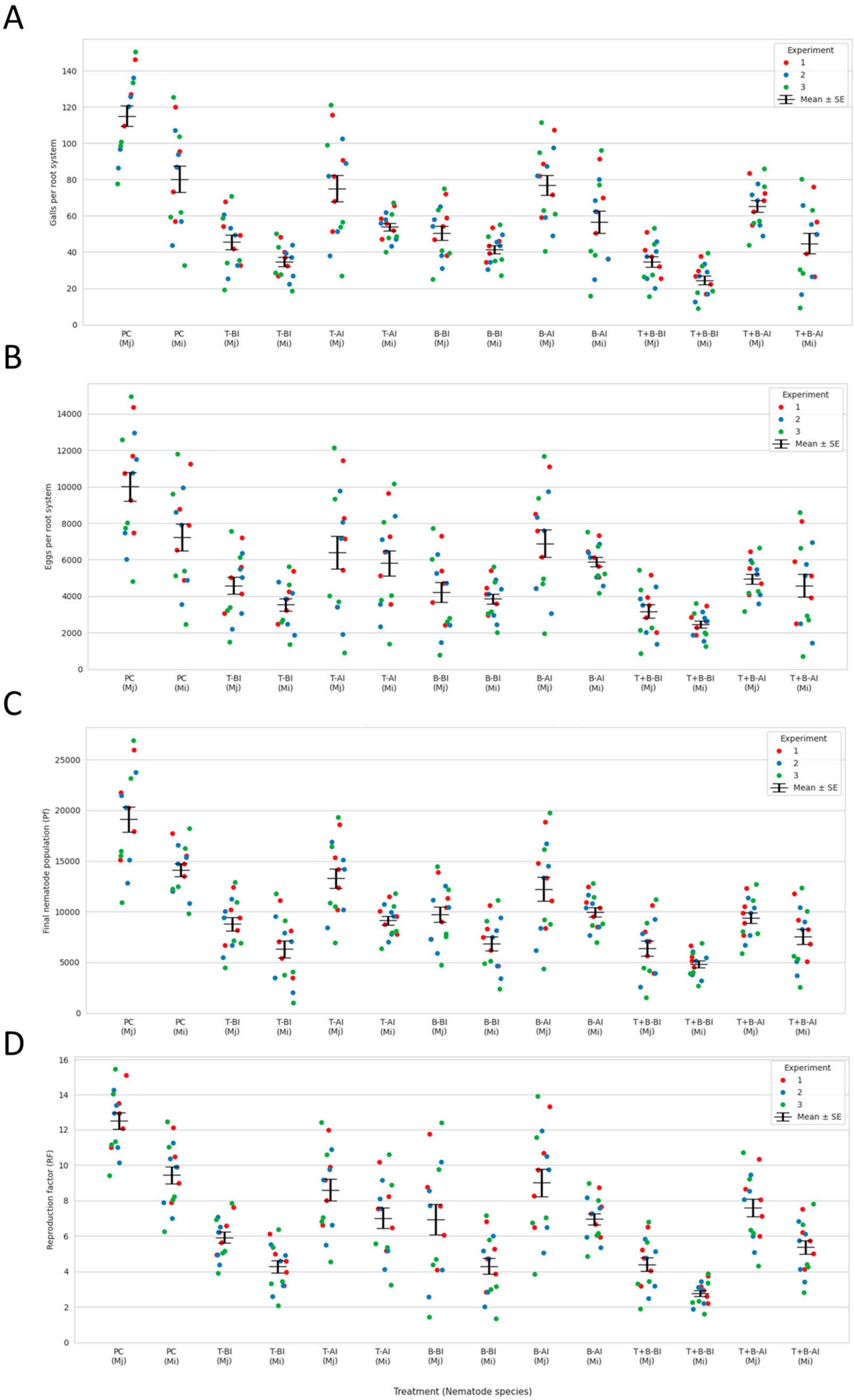
A
Galls per root system
B
Eggs per root system
C
Final nematode population (Pf)
D
Reproduction factor (RF)
Experiment
1
2
3
Mean ± SE
PC (Mj)
PC (Mi)
T-BI (Mj)
T-BI (Mi)
T-AI (Mj)
T-AI (Mi)
B-BI (Mj)
B-BI (Mi)
B-AI (Mj)
B-AI (Mi)
T+B-BI (Mj)
T+B-BI (Mi)
T+B-AI (Mj)
T+B-AI (Mi)
Treatment (Nematode species)

Figure S1. Scatter plots of individual replicate values per experimental run for nematode infestation parameters across treatments and nematode species. **(A)** galls per root system, **(B)** eggs per root system, **(C)** final nematode population (Pf), and **(D)** reproduction factor (RF). Points represent individual biological replicates (n = 5 per experiment) color-coded by experimental run (blue = Exp. 1, orange = Exp. 2, green = Exp. 3). Black structures with error bars indicate the grand mean ± SEM of the **3 experiments** ($\sum Exp_{mean} / 3$) where $Exp_{mean} = \sum$replicate values of each experiment / 5. Abbreviations stand for: PC = Positive Control (nematodes only); T–BI = *Trichoderma* applied before nematode inoculation; T–AI = *Trichoderma* applied after nematode inoculation; B–BI = *Bacillus* applied before nematode inoculation; B–AI = *Bacillus* applied after nematode inoculation; T+B–BI = *Trichoderma* + *Bacillus* applied before nematode inoculation; T+B–AI = *Trichoderma* + *Bacillus* applied after nematode inoculation. Mj: *Meloidogyne javanica*; and Mi: *M. incognita.*

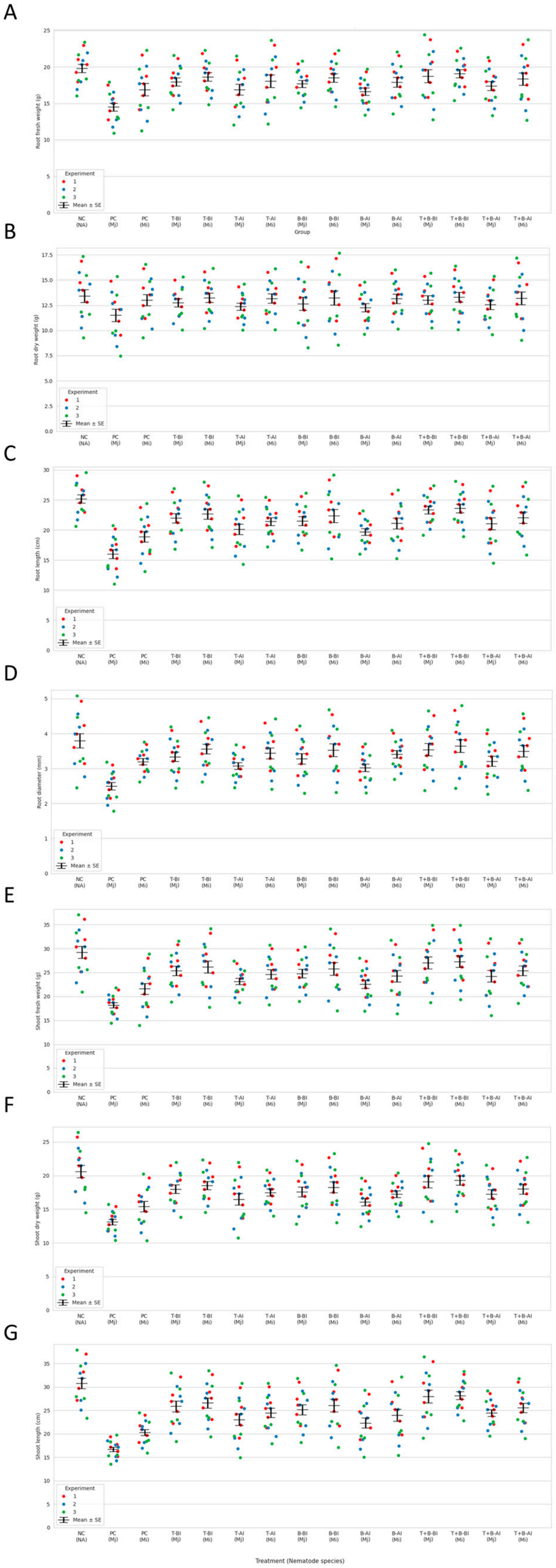
A
Root fresh weight (g)
Group
B
Root dry weight (g)
C
Root length (cm)
D
Root diameter (mm)
E
Shoot fresh weight (g)
F
Shoot dry weight (g)
G
Shoot length (cm)
Experiment
1
2
3
Mean ± SE
NC (NA)
PC (Mj)
PC (Mi)
T-BI (Mj)
T-BI (Mi)
T-AI (Mj)
T-AI (Mi)
B-BI (Mj)
B-BI (Mi)
B-AI (Mj)
B-AI (Mi)
T+B-BI (Mj)
T+B-BI (Mi)
T+B-AI (Mj)
T+B-AI (Mi)
Treatment (Nematode species)

Figure S2. Scatter plots of individual replicate values per experimental run for plant growth parameters under different nematode–biocontrol treatments. **(A)** root fresh weight (RFW), **(B)** root dry weight (RDW), **(C)** root length (RL), **(D)** root diameter (RD), **(E)** shoot fresh weight (SFW), **(F)** shoot dry weight (SDW), and **(G)** shoot length (SL). Points represent individual biological replicates (n = 5 per experiment) color-coded by experimental run (blue = Exp. 1, orange = Exp. 2, green = Exp. 3). Black structures with error bars indicate the grand mean ± SEM of the 3 experiments ($\sum Exp_{mean} / 3$) where $Exp_{mean} = \sum$replicate values of each experiment / 5. Abbreviations stand for: PC = Positive Control (nematodes only); T–BI = *Trichoderma* applied before nematode inoculation; T–AI = *Trichoderma* applied after nematode inoculation; B–BI = *Bacillus* applied before nematode inoculation; B–AI = *Bacillus* applied after nematode inoculation; T+B–BI = *Trichoderma* + *Bacillus* applied before nematode inoculation; T+B–AI = *Trichoderma* + *Bacillus* applied after nematode inoculation. Mj: *Meloidogyne javanica*; and Mi: *M. incognita.*

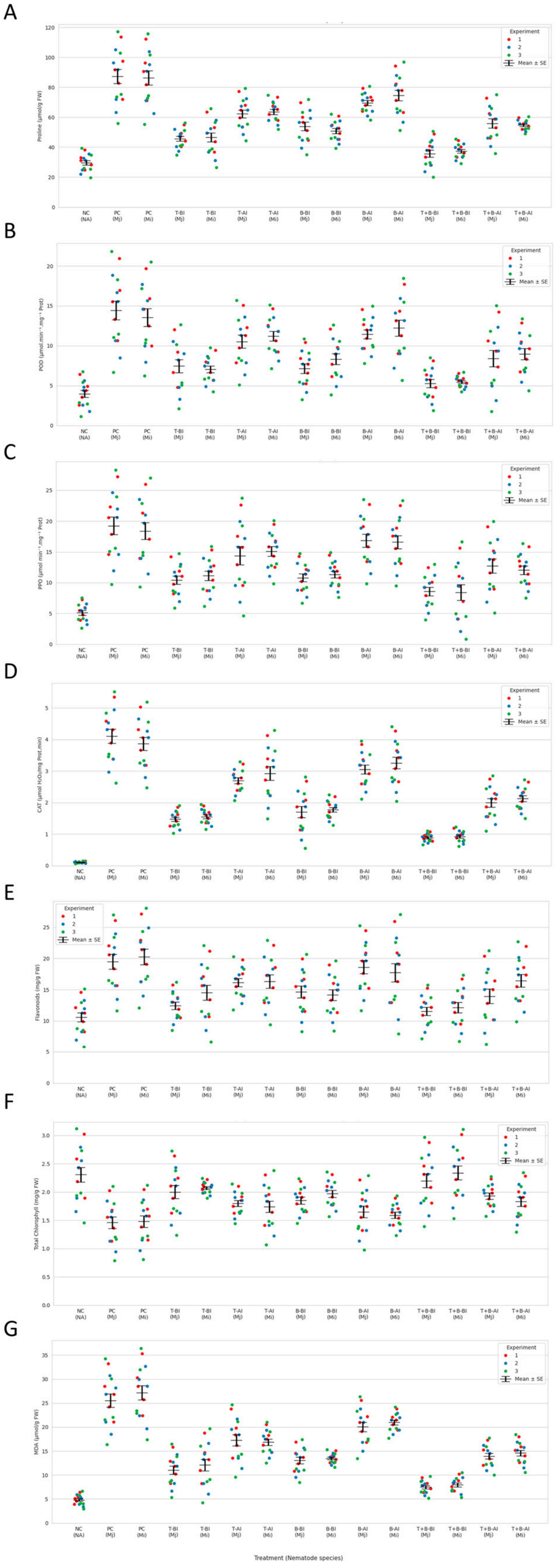
A
B
C
D
E
F
G
Proline (µmol/g FW)
POD (µmol.min⁻¹.mg⁻¹ Prot)
PPO (µmol.min⁻¹.mg⁻¹ Prot)
CAT (µmol H2O2/mg Prot.min)
Flavonoids (mg/g FW)
Total Chlorophyll (mg/g FW)
MDA (µmol/g FW)
Experiment
1
2
3
Mean ± SE
NC (NA)
PC (Mj)
PC (Mi)
T-BI (Mj)
T-BI (Mi)
T-AI (Mj)
T-AI (Mi)
B-BI (Mj)
B-BI (Mi)
B-AI (Mj)
B-AI (Mi)
T+B-BI (Mj)
T+B-BI (Mi)
T+B-AI (Mj)
T+B-AI (Mi)
Treatment (Nematode species)

Figure S3. Scatter plots of individual replicate values per experimental run for biochemical markers across biocontrol treatments used against root knot nematodes. **(A)** proline content (PRO), **(B)** peroxidase activity (POD), **(C)** polyphenol oxidase activity (PPO), **(D)** catalase activity (CAT), **(E)** flavonoid content (FLA), **(F)** total chlorophyll content (CHLT), and **(G)** malondialdehyde content (MDA). Points represent individual biological replicates (n = 5 per experiment) color-coded by experimental run (blue = Exp. 1, orange = Exp. 2, green = Exp. 3). Black structures with error bars indicate the grand mean ± SEM of the 3 experiments ($\sum Exp_{mean} / 3$) where $Exp_{mean} = \sum$replicate values of each experiment / 5. Abbreviations stand for: PC = Positive Control (nematodes only); T–BI = *Trichoderma* applied before nematode inoculation; T–AI = *Trichoderma* applied after nematode inoculation; B–BI = *Bacillus* applied before nematode inoculation; B–AI = *Bacillus* applied after nematode inoculation; T+B–BI = *Trichoderma* + *Bacillus* applied before nematode inoculation; T+B–AI = *Trichoderma* + *Bacillus* applied after nematode inoculation. Mj: *Meloidogyne javanica*; and Mi: *M. incognita.*
